# Excess Mortality Attributable to Hospital-Acquired Antimicrobial-Resistant Infections: A 2-Year Prospective Surveillance Study in Northeast Thailand

**DOI:** 10.1093/ofid/ofac305

**Published:** 2022-06-20

**Authors:** Cherry Lim, Prapit Teparrukkul, Somboon Nuntalohit, Somsamai Boonsong, Jiraphorn Nilsakul, Pramot Srisamang, Benn Sartorius, Nicholas J White, Nicholas P J Day, Ben S Cooper, Direk Limmathurotsakul

**Affiliations:** Mahidol Oxford Tropical Medicine Research Unit, Faculty of Tropical Medicine, Mahidol University, Bangkok, Thailand; Centre for Tropical Medicine and Global Health, Nuffield Department of Medicine, University of Oxford, Oxford, United Kingdom; Department of Medicine, Sunpasitthiprasong Hospital, Ubon Ratchathani, Thailand; Infectious Disease Control Department, Sunpasitthiprasong Hospital, Ubon Ratchathani, Thailand; Infectious Disease Control Department, Sunpasitthiprasong Hospital, Ubon Ratchathani, Thailand; Microbiology Laboratory, Sunpasitthiprasong Hospital, Ubon Ratchathani, Thailand; Department of Pediatrics, Sunpasitthiprasong Hospital, Ubon Ratchathani, Thailand; Centre for Tropical Medicine and Global Health, Nuffield Department of Medicine, University of Oxford, Oxford, United Kingdom; Department of Health Metrics Sciences, School of Medicine, University of Washington, Seattle, Washington, USA; Mahidol Oxford Tropical Medicine Research Unit, Faculty of Tropical Medicine, Mahidol University, Bangkok, Thailand; Centre for Tropical Medicine and Global Health, Nuffield Department of Medicine, University of Oxford, Oxford, United Kingdom; Mahidol Oxford Tropical Medicine Research Unit, Faculty of Tropical Medicine, Mahidol University, Bangkok, Thailand; Centre for Tropical Medicine and Global Health, Nuffield Department of Medicine, University of Oxford, Oxford, United Kingdom; Mahidol Oxford Tropical Medicine Research Unit, Faculty of Tropical Medicine, Mahidol University, Bangkok, Thailand; Centre for Tropical Medicine and Global Health, Nuffield Department of Medicine, University of Oxford, Oxford, United Kingdom; Mahidol Oxford Tropical Medicine Research Unit, Faculty of Tropical Medicine, Mahidol University, Bangkok, Thailand; Centre for Tropical Medicine and Global Health, Nuffield Department of Medicine, University of Oxford, Oxford, United Kingdom; Department of Tropical Hygiene, Faculty of Tropical Medicine, Mahidol University, Bangkok, Thailand

**Keywords:** antimicrobial resistance, excess mortality, hospital-acquired infection, nosocomial infection

## Abstract

**Background:**

Quantifying the excess mortality attributable to antimicrobial-resistant (AMR) bacterial infections is important for assessing the potential benefit of preventive interventions and for prioritization of resources. However, there are few data from low- and middle-income countries.

**Methods:**

We conducted a 2-year prospective surveillance study to estimate the excess mortality attributable to AMR infections for all types of hospital-acquired infection (HAI), and included bacterial species that were both locally relevant and included in the World Health Organization priority list. Twenty-eight-day mortality was measured. Excess mortality and population attributable fraction (PAF) of mortality caused by AMR infections compared to antimicrobial-susceptible (AMS) infections, adjusted for predefined confounders, were calculated.

**Results:**

We enrolled 2043 patients with HAIs. The crude 28-day mortality of patients with AMR and AMS infections was 35.5% (491/1385) and 23.1% (152/658), respectively. After adjusting for prespecified confounders, the estimated excess mortality attributable to AMR infections was 7.7 (95% confidence interval [CI], 2.2–13.2) percentage points. This suggests that 106 (95% CI, 30–182) deaths among 1385 patients with AMR infections might have been prevented if all of the AMR infections in this study were AMS infections. The overall PAF was 16.3% (95% CI, 1.2%–29.1%). Among the bacteria under evaluation, carbapenem-resistant *Acinetobacter baumannii* was responsible for the largest number of excess deaths. Among all types of infection, urinary tract infections were associated with the highest number of excess deaths, followed by lower respiratory tract infections and bloodstream infections.

**Conclusions:**

Estimating and monitoring excess mortality attributable to AMR infections should be included in national action plans to prioritize targets of preventive interventions.

**Clinical Trials Registration:**

NCT03411538.

## Teaser

Burden of antimicrobial-resistant (AMR) bacterial infection is high in low- and middle-income countries. Data generated from prospective surveillance on multiple organisms and for every type of hospital-acquired infection could support prioritizing targets of preventive interventions to control the spread of AMR infection.

Quantification of the health burden related to antimicrobial resistance (AMR) is essential to understand the extent of the health impact, to improve management, and to allocate resources accordingly [1, 2]. Differentiating hospital-acquired infection (HAI) from community-acquired infections is important for designing the appropriate interventions, as the epidemiology of pathogenic bacteria and patient characteristics of the 2 infections can be different. However, quality data from low- and middle-income countries (LMICs) on HAIs are limited. Conversion factors that describes the relative mortality of each type of infection, such as lower respiratory tract infections (LRTIs), compared to bloodstream infections (BSIs) have been used to estimate the overall burden of AMR infections when only BSI data are available on several occasions [1, 3–5]. In 2015, the review on AMR led by Jim O’Neill estimated that 700 000 deaths could be caused each year by AMR bacterial infections, including multidrug-resistant and extensive drug-resistant tuberculosis, globally [1, 6]. There were limited data from LMICs at that time [1, 7], and the deaths attributable to AMR infections in LMICs were estimated based on conversion factors from United States (US) data in 2013 [4, 8]. The European Centre for Disease Prevention and Control (ECDC) and the European Medicines Agency estimated that 25 000 deaths were attributable to AMR infections in European countries in 2009 [4]. This number was updated to 33 110 (95% uncertainty interval: 28 480–38 430) deaths in 2018 by Cassini et al [9] based on systematic reviews, local data on blood culture results, and conversion factors. A study from Thailand in 2016 estimated that 19 000 deaths were attributable to multidrug-resistant HAIs [5]. Conversion factors were again used to generate the estimate [5]. Recently, the Global Burden of Disease (GBD) Study estimated that 1.27 million deaths were attributable to AMR bacterial infections globally in 2019 [10]. Global and regional estimates of AMR burden depend heavily on modeling assumptions as detailed clinical and microbiology data are scarce [11–13].

To bridge the gap of missing quality data on the relative mortality of each type of AMR infections in LMICs, we conducted a prospective surveillance study with an objective to estimate excess mortality and deaths attributable to AMR infections compared to antimicrobial-susceptible (AMS) infections for all types of HAIs in a large public hospital in northeast Thailand.

## METHODS

### Study Design

We prospectively enrolled patients who were admitted to Sunpasitthiprasong Hospital, a 1201-bed tertiary hospital in Ubon Ratchathani, northeast Thailand. The most commonly used parenteral antibiotics in this hospital were ceftriaxone, carbapenems, and ceftazidime [14]. All wards and patients of all ages were screened. The inclusion criteria were patients, regardless of age or ward at which treatment was received, who developed BSI, LRTI, surgical site infection (SSI), urinary tract infection (UTI), or other specific types of infections (OTH) after hospitalization for >48 hours with clinical specimens culture positive for *Staphylococcus aureus*, *Enterococcus* spp, *Escherichia coli*, *Klebsiella pneumoniae*, *Pseudomonas aeruginosa*, or *Acinetobacter* spp. Protocol training and a “dry run” were performed between January and February 2018, before the surveillance study was rolled out between 1 March 2018 and 29 February 2020.

### Definitions

The definitions of BSI, LRTI, SSI, UTI, and OTH were based on the US Centers for Disease Control and Prevention surveillance guidelines [15], the Infectious Diseases Society of America/American Thoracic Society guidelines for pneumonia [16], and the ECDC point-prevalence survey study [17]. Detailed definitions used for each infection are given in Supplementary Appendix 1.

Infections with methicillin-resistant *S aureus* (MRSA), ampicillin-resistant *Enterococcus faecium* (AMPREfm), *Enterococcus faecalis* (AMPREfc), third-generation cephalosporin-resistant *E coli* and *K pneumoniae* (3GCREC and 3GCRKP), and carbapenem-resistant *P aeruginosa* and *Acinetobacter baumannii* (CRPA and CRAB) are defined as AMR infections. Infections caused by bacteria that were susceptible to the corresponding antibiotic of interest are defined as AMS infections. Polymicrobial infections are defined as infections with >1 of the bacteria under evaluation. AMPREfm and AMPREfc were chosen rather than vancomycin-resistant *E faecium* (VREfm) [15] because VREfm was very rare in our setting (n = 11); moreover, association in AMPREfm and AMPREfc with high mortality has been reported in other settings [18]. Polymicrobial infection with at least 1 AMR pathogen is defined as AMR infection.

Clinical severity scores including Sequential Organ Failure Assessment (SOFA) score and the Charlson Comorbidity Index (CCI) score were recorded. Detailed definitions of the comorbidities and severity scores are shown in Supplementary Appendix 2.

### Statistical Analysis

The excess mortality and population attributable fraction (PAF) of mortality caused by AMR infections compared to AMS infections was estimated as described previously [19–21]. Detailed descriptions of the statistical analysis are shown in Supplementary Appendix 3. In short, as 28-day mortality was recorded regardless of whether or not patients were discharged from the hospital before observing the outcome, discharge was not a competing outcome of the 28-day mortality in this study. Hence, logistic regression models were used to estimate the impact of AMR infections on 28-day mortality. Multivariable logistic regression models were built based on a direct acyclic graph, which is presented in Supplementary Appendix 4. The predefined confounders were (1) the severity of underlying illness (ie, measured by CCI score and SOFA scores) and patient characteristics on the day of hospital admission, and (2) factors that may increase risks of acquiring AMR infections during the hospitalization. Bacterial load is a strong risk factor to determine mortality and was not directly quantified for every patient in this study; hence, turnaround time for bacterial culture result was used as a proxy for bacterial load (Supplementary Appendix 2). The excess risk of mortality is defined as the absolute mortality in the study cohort that would have been prevented if patients had AMS infections. PAF is defined as the proportional reduction in population mortality that would be prevented if all patients had AMS infections, adjusted for predefined confounders [19–22].

Sensitivity analysis was performed to assess the impact of the patients who were lost to follow-up had on the estimated attributable mortality. All statistical analyses were done using Stata, version 17.0 (StataCorp LP, College Station, Texas).

### Ethical Approval

The study was approved by the ethics committee of the Faculty of Tropical Medicine, Mahidol University, Thailand (reference number TMEC 17-057) and by the ethics committee of Sunpasitthiprasong Hospital, Ubon Ratchathani, Thailand (reference number 092/2560). Details on the informed consent process performed can be found in Supplementary Appendix 3. The study was registered at ClinicalTrials.gov (NCT03411538).

### Patient Consent Statement

Signed or fingerprinted informed written consent was obtained from patients or their representatives before enrollment. The study was conducted in full compliance with the principles of good clinical practice and the ethical principles of the Declaration of Helsinki. The study protocol and related documents were approved by the ethics committee of the Faculty of Tropical Medicine, Mahidol University, Thailand (reference number TMEC 17-057) and by the ethics committee of Sunpasitthiprasong Hospital, Ubon Ratchathani, Thailand (reference number 092/2560).

## RESULTS

### Patient Population

**Figure 1.**
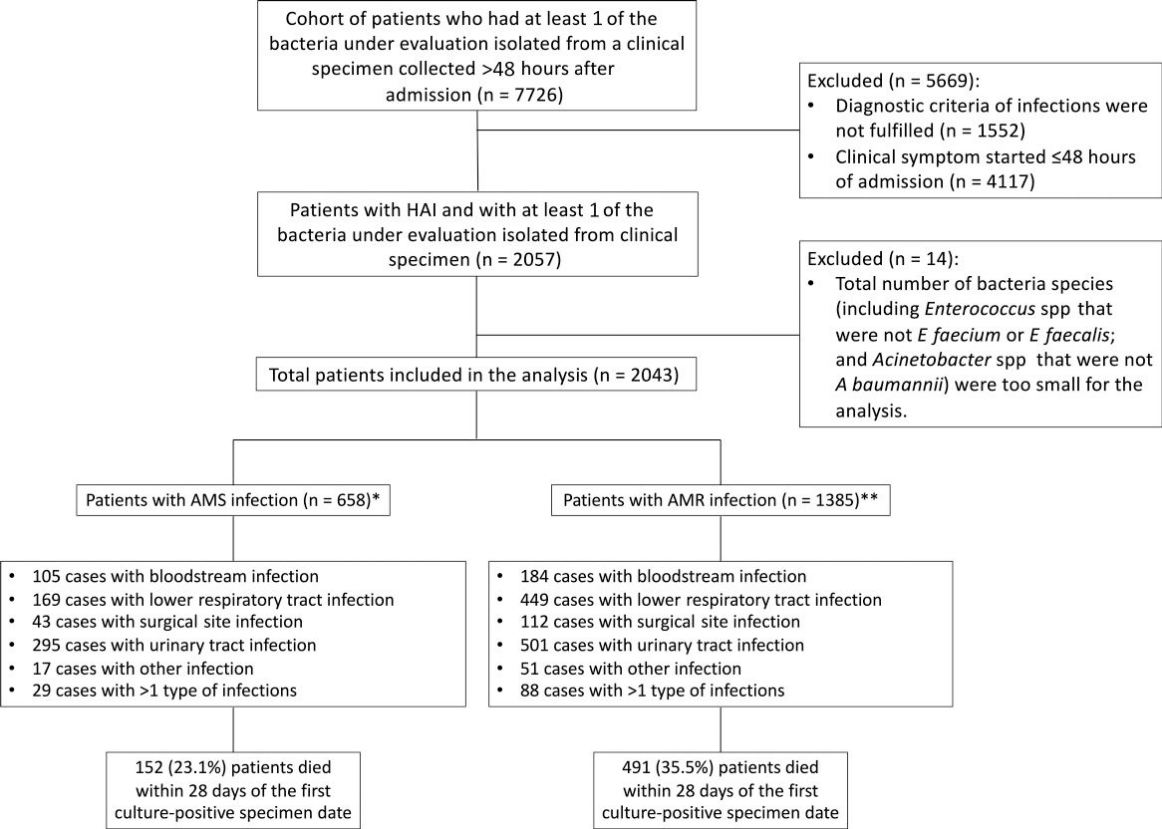
Study flow diagram. Infections with methicillin-resistant *Staphylococcus aureus*, ampicillin-resistant *Enterococcus faecium* and *Enterococcus faecalis*, third-generation cephalosporin-resistant *Escherichia coli* and *Klebsiella pneumoniae*, and carbapenem-resistant *Pseudomonas aeruginosa* and *Acinetobacter baumannii* are defined as antimicrobial-resistant infections. Infections caused by the bacteria that was susceptible to the corresponding antibiotic of interest are defined as antimicrobial-susceptible (AMS) infections. *Of the 658 patients with AMS infection, 11 were lost to follow-up. Among these 11 patients, 5 were discharged against medical advice with health condition not improving and were assumed to have died within 28 days of follow-up since the first date of culture-positive specimen collection. **Of the 1385 patients, 28 were lost to follow-up. Among these 28 patients, 23 were discharged against medical advice with health condition not improving, and were assumed to have died within 28 days of follow-up since the first date of culture-positive specimen collection. Abbreviations: AMR, antimicrobial-resistant; AMS, antimicrobial-susceptible; HAI, hospital-acquired infection.

Over the 2-year study period, bacterial culture results (both negative and positive culture results) were received from 282 247 clinical samples (with an average of 378 clinical samples per day) collected from 54 281 patients. Of the 282 247 clinical samples, 36%, 13%, 34%, and 17% were blood, urine, respiratory tract specimens, and other clinical samples, respectively. The study team checked the eligibility criteria for a total of 7726 patients who had at least 1 of the bacteria under evaluation isolated from a clinical specimen collected >48 hours after admission (Figure 1). Of the 7726 patients, 4117 were excluded because an onset of signs and symptoms of the current infections were documented within the first 48 hours of hospital admission, and 1552 were excluded because the diagnostic criteria of LRTI, SSI, UTI, or OTH were not fulfilled. Of all patients screened, 2057 patients who fulfilled the criteria of hospital-acquired BSI, LRTI, SSI, UTI, or OTH were enrolled. We further excluded 14 patients from the analysis because the total number of patients from whom a particular bacterial species was isolated was too small for the analysis (including *Enterococcus* spp that were not *E faecium* or *E faecalis* [n = 1] and *Acinetobacter* spp that were not *A baumannii* [n = 13]). In total, 2043 patients were included in the analysis. Of the 2043 patients, 39 patients were lost to follow-up and, among these patients, 28 were discharged against medical advice with a serious medical condition that had not improved and hence were assumed to have died within 28 days of follow-up since the first date of culture-positive specimen collection. Separately, sensitivity analyses were performed where these 39 patients were assumed to have died within 28 days of follow-up and where the patients were assumed to have survived within 28 days of follow-up.

Of the 2043 patients, 1385 (67.8%) had infections with at least 1 of the AMR bacteria under evaluation; they were classified as having AMR infections (Table 1). Of the 2043 patients, 1163 (56.9%) were males, and the median age was 61 years (interquartile range [IQR], 45–72 years). The median length of hospital stay prior to the first date of culture-positive specimen collection was 11 days (IQR, 7–19 days), and was longer for the AMR infection group (median, 14 days [IQR, 8–22 days]) compared to the AMS infection group (median, 8 days [IQR, 6–14 days]).

**Table 1.** Characteristics of Patients Included in the Analysis

Characteristics	AMS Infections (n = 658 Patients)	AMR Infections (n = 1385 Patients)
Male sex	347 (52.7)	816 (58.9)
Age, y		
≤0	19 (2.9)	65 (4.7)
1–4	7 (1.1)	15 (1.1)
5–14	17 (2.6)	25 (1.8)
15–24	37 (5.6)	65 (4.7)
25–34	26 (4.0)	64 (4.6)
35–44	52 (7.9)	107 (7.7)
45–54	102 (15.5)	179 (12.9)
55–64	126 (19.2)	287 (20.7)
65–80	200 (30.4)	440 (31.8)
≥81	72 (10.9)	138 (10.0)
Bacteria under evaluation		
*Staphylococcus aureus*	64 (9.7)	5 (0.4)
*Enterococcus faecium*	6 (0.9)	176 (12.7)
*Enterococcus faecalis*	131 (19.9)	29 (2.1)
*Escherichia coli*	101 (15.4)	188 (13.6)
*Klebsiella pneumoniae*	73 (11.1)	267 (19.3)
*Pseudomonas aeruginosa*	140 (21.3)	81 (5.9)
*Acinetobacter baumannii*	65 (9.9)	336 (24.3)
Polymicrobial	78 (11.9)	303 (21.9)
Types of infection		
Bloodstream infection	105 (16.0)	184 (13.3)
Lower respiratory tract infection	169 (25.7)	449 (32.4)
Surgical site infection	43 (6.5)	112 (8.1)
Urinary tract infection	295 (44.8)	501 (36.2)
Other infection	17 (2.6)	51 (3.7)
>1 type of infection^a^	29 (4.4)	88 (6.4)
Admission ward		
Non-ICU medical ward	221 (33.6)	494 (35.7)
Non-ICU surgical ward	243 (36.9)	497 (35.9)
Non-ICU hematology/oncology	28 (4.3)	34 (2.5)
Non-ICU obstetrics/gynecology	10 (1.5)	9 (0.7)
ICU	156 (23.7)	351 (25.3)
Health status at time of admission		
CCI score, median (IQR)	3 (1–5)	3 (1–5)
Transferred from other hospitals	446 (67.6)	939 (67.8)
SOFA score, median (IQR)	2 (0–4)	2 (1–4)
Parenteral antibiotic usage prior to the first date of culture-positive specimen collection		
Exposure to parenteral antibiotics	553 (84.0)	1254 (90.5)
Cumulative days of exposure to parenteral antibiotics, median (IQR)	1 (1–7)	7 (1–13)
Health status on the first date of culture-positive specimen collection		
In the ICU	267 (40.6)	679 (49.0)
SOFA score, median (IQR)	1 (0–4)	2 (0–4)
Length of hospital stay		
Days in the hospital prior to specimen collection, median (IQR)	8 (6–14)	14 (8–22)
Turnaround time for bacterial culture result		
≤3 d	576 (87.5)	1171 (84.6)
>3 d	82 (12.5)	214 (15.5)
Outcome		
28-d mortality	152 (23.1)	491 (35.5)
Time to death^b^, d, median (IQR)	10.5 (4–19)	9 (3–17)
Length of hospital stay after the first date of culture-positive specimen collection in survivors, d, median (IQR)	14 (7–27)	18 (9–35)

Data are presented as No. (%) unless otherwise indicated. Infections with methicillin-resistant *Staphylococcus aureus*, ampicillin-resistant *Enterococcus faecium*, *Enterococcus faecalis*, third-generation cephalosporin-resistant *Escherichia coli* and *Klebsiella pneumoniae*, and carbapenem-resistant *Pseudomonas aeruginosa* and *Acinetobacter baumannii* are defined as AMR infections. Infections caused by the bacteria that was susceptible to the corresponding antibiotic of interest are defined as AMS infections.

Abbreviations: AMR, antimicrobial-resistant; AMS, antimicrobial-susceptible; CCI, Charlson Comorbidity Index; ICU, intensive care unit; IQR, interquartile range; SOFA, Sequential Organ Failure Assessment.

a “>1 type of infection” refers to patient who infections that occurred at ≥2 body sites.

b Statistics were estimated for those who died.

### Causative Bacteria

**Figure 2.**
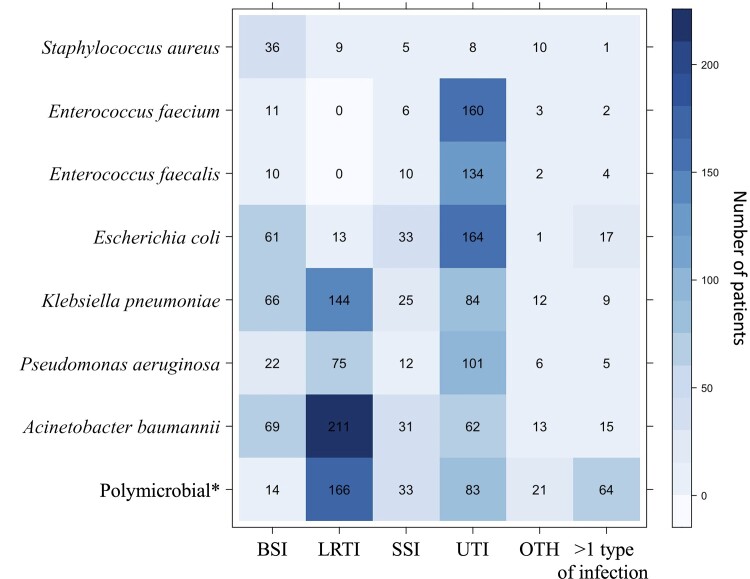
A level plot illustrating number of patients with hospital-acquired infections by each causative bacteria and type of infection. “>1 type of infection” is defined as infections that occurred with ≥2 types of infection including bloodstream infection (BSI), lower respiratory tract infection (LRTI), surgical site infection (SSI), urinary tract infection (UTI), and infections at other body sites (OTH). *Polymicrobial is defined as infections with >1 of the bacteria included in the evaluation.

Of the 2043 patients, 1662 patients (81.4%) had monomicrobial infections (ie, infections with a single bacterium of interest under this study). Among the patients with monomicrobial infections, *A baumannii* (n = 401), followed by *K pneumoniae* (n = 340) and *E coli* (n = 289), were the most commonly identified bacteria. Three hundred eighty-one (18.7%) patients had polymicrobial infection in this cohort (Figure 2).

Of all monomicrobial infections, the proportion of MRSA, AMPREfm, and AMPREfc among infections with *S aureus*, *E faecium*, and *E faecalis* were 7.2% (5/69), 96.7% (176/182), and 18.1% (29/160), respectively. The proportion of 3GCREC, 3GCRKP, CRPA, and CRAB among infections with *E coli*, *K pneumoniae*, *P aeruginosa*, and *A baumannii* were 65.1% (188/289), 78.5% (267/340), 36.7% (81/221), and 83.8% (336/401), respectively. Among infections with *E faecium*, 6.0% (11/182) were vancomycin resistant, and these 11 isolates were also resistant to ampicillin. None of the *E faecalis* infections were vancomycin resistant.

### Types of Infections

Most of the patients had infections involving a single body site (n = 1926/2043 [94.3%]). The most common type of infection was UTI (n = 796), followed by LRTI (n = 618), BSI (n = 289) with no focus of infection, SSI (n = 155), >1 type of infection (n = 117), and OTH (n = 68). Of 117 patients with >1 type of infection, 27 were secondary bacteremia related to UTI (ie, patients with matched bacteria in blood and urine samples), 48 were secondary bacteremia related to LRTI, and 6 were secondary bacteremia related to SSI.

Patient characteristics at hospital admission varied by type of infection. Patients with >1 type of infection had, on average, the highest CCI score (Supplementary Appendix 5).

### Observed Mortality

Overall, crude 28-day mortality was higher in the AMR infection group (n = 491/1385 [35.5%]) than in the AMS infection group (n = 152/658 [23.1%]). Among patients who survived up to 28 days since the first date of culture-positive specimen collection, the length of hospital stay after the first date of culture-positive specimen collection was longer in the patients with AMR infections (median, 18 days [IQR, 9–35 days]) compared to those with AMS infections (median, 13 days [IQR, 7–27 days]). Of those who died within 28 days, death occurred earlier in patients with AMR infections (median, 9 days [IQR, 4–17 days]) compared to patients with AMS infections (median, 11 days [IQR, 5–20 days]). The crude 28-day mortality by causative bacteria and type of infection is shown in Supplementary Appendix 6.

### Excess Mortality Attributable to AMR Infections

**Figure 3.**
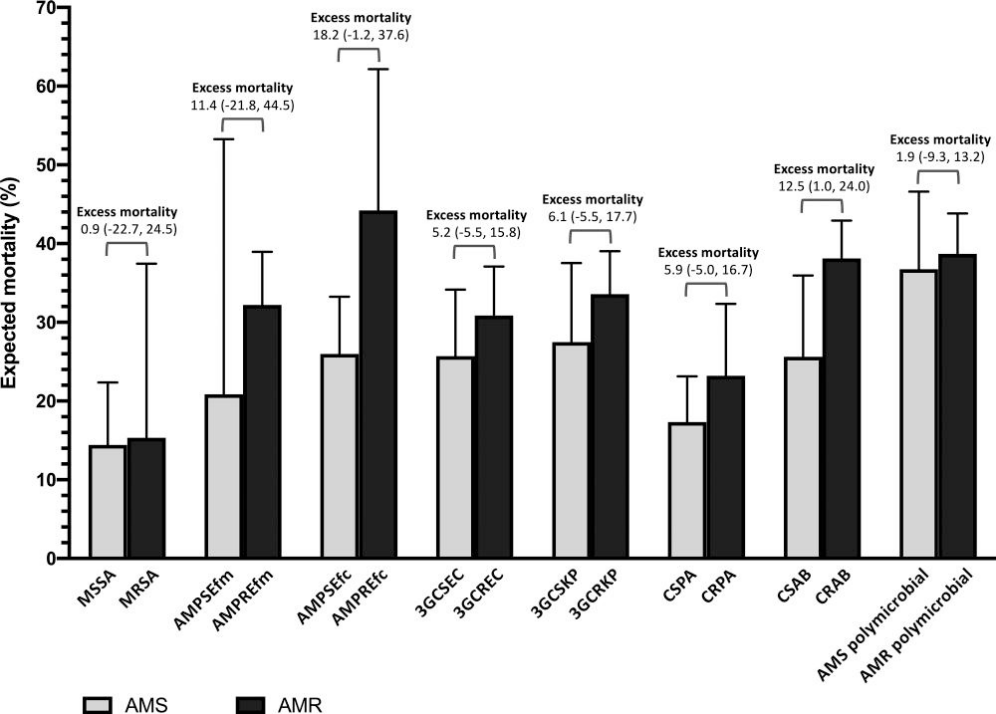
Expected mortality if all infections caused by each causative bacteria were antimicrobial susceptible (AMS; gray) or antimicrobial resistant (black). Excess mortality (percentage points; 95% confidence interval) is defined as the mortality in the study cohort that would be prevented if the infections of the specific type were AMS infections, adjusted for the predefined confounding factors. Polymicrobial is defined as infections with >1 of the bacteria in the evaluation. Abbreviations: 3GCREC, third-generation cephalosporin-resistant *Escherichia coli*; 3GCRKP, third-generation cephalosporin-resistant *Klebsiella pneumoniae*; 3GCSEC, third-generation cephalosporin-susceptible *Escherichia coli*; 3GCSKP, third-generation cephalosporin-susceptible *Klebsiella pneumoniae*; AMPREfc, ampicillin-resistant *Enterococcus faecalis*; AMPREfm, ampicillin-resistant *Enterococcus faecium*; AMPSEfc, ampicillin-susceptible *Enterococcus faecalis*; AMPSEfm, ampicillin-susceptible *Enterococcus faecium*; AMR, antimicrobial resistant; AMS, antimicrobial-susceptible; CRAB, carbapenem-resistant *Acinetobacter baumannii*; CRPA, carbapenem-resistant *Pseudomonas aeruginosa*; CSAB, carbapenem-susceptible *Acinetobacter baumannii*; CSPA, carbapenem-susceptible *Pseudomonas aeruginosa*; MRSA, methicillin-resistant *Staphylococcus aureus*; MSSA, methicillin-susceptible *Staphylococcus aureus.*

**Figure 4.**
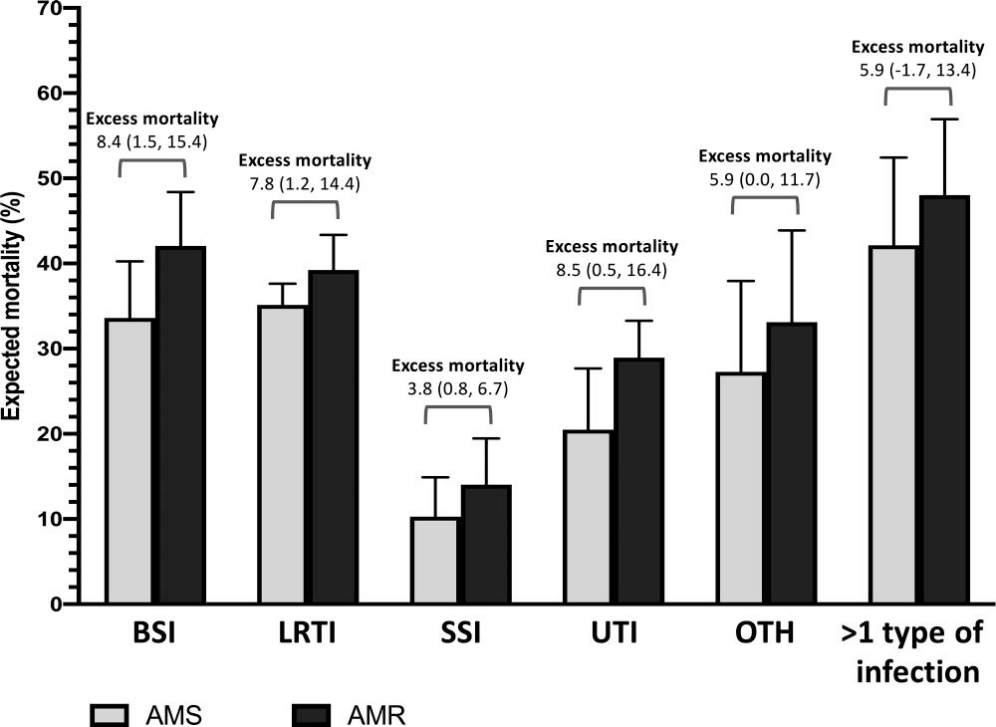
Expected mortality if all infections for each type of infection were antimicrobial susceptible (AMS; gray) or antimicrobial resistant (black). Excess mortality (percentage points; 95% confidence interval) is defined as the mortality in the study cohort that would be prevented if the infections of the specific type were AMS infections, adjusted for the predefined confounding factors. Abbreviations: AMR, antimicrobial-resistant; AMS, antimicrobial-susceptible; BSI, bloodstream infection; LRTI, lower respiratory tract infection; OTH, other infections; SSI, surgical site infection; UTI, urinary tract infection; >1 type of infection, infections that occurred with ≥2 types including BSI, LRTI, SSI, UTI, and OTH.

The model that included an interaction term between the bacterial species and AMR infections and adjusted for all predefined confounding factors was used to estimate excess mortality attributable to AMR infections. We estimated that if all of the infections were AMS infections, the mortality would have been 26.3% (95% confidence interval [CI], 21.7%–31.0%), and if all of the infections were AMR infections, the mortality would have been 34.0% (95% CI, 31.3%–36.7%). This represented an absolute difference of 7.7 (95% CI, 2.2–13.2) percentage points of excess mortality attributable to AMR infections. The estimated PAF of mortality caused by AMR infections was 16.3% (95% CI, 1.2%–29.1%), which implied that approximately 106 deaths of the 643 observed deaths could be prevented if all individuals in the study cohort had AMS infections. Figures 3 and 4 illustrate expected mortality if all infections were AMS or AMR by causative organism and type of infection, respectively. Of all AMR bacteria under evaluation, CRAB accounted for the highest number of excess deaths attributable to AMR infections (42 deaths [95% CI, 3–81]), followed by AMPREfm and 3GCRKP (Supplementary Appendix 7). Of all types of infection under evaluation, UTI accounted for the highest number of excess deaths attributable to AMR infections (42 deaths [95% CI, 3–82]). This was followed by LRTI (35 deaths [95% CI, 5–65]) and BSI (16 deaths [95% CI, 3–28]; Supplementary Appendix 8). Results from the sensitivity analyses are shown in Supplementary Appendix 9, and results of the multivariable logistic regression model are shown in Supplementary Appendix 10.

## DISCUSSION

In this study, we estimated overall mortality attributable to AMR infections, and mortalities attributable to AMR infections for each causative bacteria and for each type of HAI in a large public hospital in northeast Thailand. The excess mortality attributable to AMR infections in this study cohort is 7.7 percentage points (ie, 77 per 1000 patients with HAI) and the overall PAF is 16.3% (ie, 106 preventable deaths of 643 observed deaths). This is relatively higher than that reported from high-income countries (HICs). Of the AMR bacteria under evaluation, CRAB, AMPREfm, and 3GCRKP had the largest number of excess deaths. Of types of infections under evaluation, UTI, LRTI, and BSI had the largest number of excess deaths. This suggests that the local action plans on AMR would need to focus on those infections and develop interventions that can reduce the burden of AMR related to those infections effectively.

Comparisons between estimates from our cohort and other studies in LMICs and HICs are difficult because of differences in study population, study pathogens, study designs, clinical procedures and practices, and comparators and estimators reported. Our estimates are also not directly comparable with the burden of AMR infections in the European Union reported by Cassini et al [9] in 2019 and globally reported by the GBD study in 2021 [10] because neither overall excess mortality nor PAF of mortality caused by AMR infections were explicitly reported in those studies. Nonetheless, Cassini et al estimated that the total number of patients with GCREC, GCRKP, MRSA, CRPA, and - carbapenem-resistant *Acinetobacter* spp infections in the European Union and European Economic Area was 603 966 (based on result table of the article) and the estimated number of deaths attributable to those AMR infections was 26 320. Therefore, with post hoc estimation, excess mortality attributable to those AMR infections is about 4.4% (26 320/603 966). In the GBD study [10], the estimated number of patients with GCREC, GCRKP, MRSA, CRPA, and CRAB infections globally was 14 432 680, and the estimated number of deaths attributable to those AMR infections was 326 800. The excess mortality attributable to those AMR infections in the GBD study is about 2.5% (326 800/14 432 680). In our study, the observed total number of patients with GCREC, GCRKP, MRSA, CRPA, and CRAB infections was 877, and the estimated number of deaths attributable to those AMR infections was 74. The excess mortality attributable to those AMR infections in our study is about 8.4% (74/877), which was relatively higher than that estimated in the European Union (4.4%) and the global estimate in the GBD study (2.5%). The estimated excess mortality in this study cohort represents the lower limit of the impact of AMR in our setting as we compared mortality of patients with AMR and AMS infections [2].

An increasing number of studies are reporting the importance of CRAB, AMPREfm, and 3GCRKP organisms. CRAB and 3GCRKP are priority AMR bacteria accounting for high excess mortality and deaths attributable to AMR infections in Europe [11], US [13], Greece [23], and Thailand [5]. Vancomycin-resistant *Enterococcus* is a priority AMR bacteria in HICs [9, 13, 23] but is not commonly found in our setting. Most patients with *Enterococcus* infections were patients with UTI, followed by BSI, in our setting. Evidence of the impact of AMPREfm infections remains scarce. A study of *E faecium* bacteremia in Spain reported an overall mortality of 34% and 21% in patients with AMPREfm and AMPSEfm bacteremia, respectively [24]. A study in Tanzania also raises a concern about ampicillin-resistant enterococcal infections and their treatment in Africa [25]. It is possible that the excess mortality associated with ampicillin-resistant enterococcal infections observed in our study could be partly due to residual confounding. The wide 95% CI around the estimate implied that the study did not have sufficient power to estimate its impact precisely. Further studies are needed to provide more evidence on the impact of AMR UTI and AMPREfm in our setting and other LMICs. This would be important to guide diagnosis, treatment, and practice of preventing hospital-acquired UTI, LRTI, and BSI in LMICs.

An important strength of this study is that we prospectively collected detailed patient information allowing us to adjust for important confounders associated with mortality due to AMR infections systematically [26]. We also examined the epidemiology of AMR infections for different types of infection and a range of bacteria in a complete cohort. This allowed us to estimate the overall burden of AMR infection, and the burden of AMR by types of infections and by AMR bacteria systematically. We recorded the 28-day outcome for almost all patients enrolled in the study.

There are several limitations to this study, as with many studies addressing this important question. First there are important residual confounding factors. For example, antibiotic use before hospital admission is common but often not recorded. Second, some important pathogens (*S aureus*, *Acinetobacter* spp) could colonize skin and could be incidental in blood cultures. Third, excess mortality and deaths attributable to AMR infections compared to AMS infections represents the lower bound of burden of AMR infections, and may underestimate potential impacts of future interventions and preventive measures against AMR infections [2, 27]. An additional analysis to estimate the excess deaths of AMR infections compared to patients with no infections will be needed to inform the upper bound of burden of AMR infections [2, 27]. Fourth, as the study was performed in a single study site and Thailand is an upper-middle-income country, the results may not be generalizable to all settings where patient characteristics, patient management, antibiotic availability and usage, and practices of collecting clinical samples for microbiology culture (including frequency and timing) may differ from the study hospital. However, the study design can be readily adapted and applied in other LMICs with a relative high sustainability to produce detailed information to support target organism(s) and type(s) of infection to intervene. Subsequent episodes of HAI prior 28-day outcome could be an important mediator of the first episode of AMR and 28-day outcome, and further studies with appropriate analysis could be done to quantify the impact of subsequent episodes on patient outcome. Finally, the appropriateness of antibiotic use could be an important mediator of AMR infections and mortality. Further studies with appropriate adjustment for immortal-time bias and time-varying confounders could be done to disentangle the relationships and improve the understanding of the mechanism underlying the impact of AMR [28].

## Supplementary Material

ofac305_Supplementary_Data

## Supplementary Data

Supplementary materials are available at *Open Forum Infectious Diseases* online. Consisting of data provided by the authors to benefit the reader, the posted materials are not copyedited and are the sole responsibility of the authors, so questions or comments should be addressed to the corresponding author.

## Notes

***Author contributions.*** C. L., D. L., and P. T. had full access to the data described in the study. The corresponding author has the final decision to submit for publication.

***Acknowledgments.*** We thank Kanthiya Jirapornuwat, Rampaipan Wongwattanasatean, Gumphol Wongsuvan, Areeya Faosap, Yaowaret Dokket, Sukhumal Pewlaorng, Jintana Suwannapruek, and Prapass Wannapinij for their clinical, laboratory, and administrative support. We gratefully acknowledge the support provided by staff at the Sunpasitthiprasong Hospital and at the Mahidol Oxford Tropical Medicine Research Unit.

***Open access.*** For the purpose of open access, the author has applied a CC BY public copyright license to any author-accepted manuscript version arising from this submission.

***Data availability.*** The data that support the findings of this study are available on request.

***Disclaimer.*** The funders of the investigators and study had no role in the study design, data collection, data analysis, data interpretation, or writing of the manuscript.

***Financial support***. C. L. is funded by a Wellcome Trust Research Training Fellowship (grant number 206736/Z/17/Z). D. L. is funded by a Wellcome Trust Intermediate Training Fellowship (grant number 101103). B. S. C. is funded by the UK Medical Research Council and UK Department for International Development (grant number MR/K006924/1). This study was also supported by the Wellcome Trust as part of the Wellcome Trust–Mahidol Oxford Tropical Medicine Research Programme (grant number 106698/Z/14/Z). B. S is supported by a grant from the UK Department of Health and Social Care using UK aid funding managed by the Fleming Fund (R52354 CN001).
