# Supplementary material for: Excess Mortality Attributable to Hospital-Acquired Antimicrobial-Resistant Infections: A 2-Year Prospective Surveillance Study in Northeast Thailand
Source: Open Forum Infect Dis. 2022 Jun 20;9(9):ofac305. doi: 10.1093/ofid/ofac305 (PMC9454027; doi:10.1093/ofid/ofac305)
Supplement: ofac305_Supplementary_Data [file ofac305_supplementary_data.docx]

**Appendix 1.** Definitions for types of infection.

| Type of infection | Definition |
| --- | --- |
| Bloodstream infection (BSI) | Patient had blood culture positive for at least one of the six organisms under evaluation*.  **Type of BSI**  Central line-associated BSI  A BSI patient who had central line or umbilical catheter in place for >2 calendar days prior to the date of blood collection and the line was also in place on the date of blood collection or the day before.  Primary BSI  A BSI patient who was not thought to be seeded from an infection at another body site.  Note: Secondary BSI was not included in this type of infection and was included in >1 type of infection defined below. |
| Lower-respiratory tract infection (LRTI) | Patient had at least one of the following:   - Fever (>38°C) - Leukopenia (≤4,000 WBC/mm^3^) or leukocytosis (≥12,000 WBC/mm^3^) - Altered mental status with no other recognized cause (if ≥70 years old)   **AND**  at least one of the following clinical symptoms:   - New onset of purulent sputum or change in character of sputum, or increased respiratory secretions, or increased suctioning requirements - New onset or worsening cough, or dyspnea or tachypnea - Rales/Crepitation or bronchial breath sounds, or wheezing or rhonchi - Worsening gas exchange (i.e. O_2_ desaturation [i.e. PaO_2_/FiO_2_≤240], increased oxygen requirements, or increased ventilator demand)   **AND**  imaging result suggested one of the followings   - Consolidation - Cavitation - New or progressive and persistent infiltration - Pneumatoceles, in infants ≤1 year old   **AND**  *At least one of the six organisms under evaluation isolated from lower-respiratory tract specimen. |
| Surgical site infection (SSI) | Superficial incisional SSI (Patient who had all of the following:)   - Infection occurred within 30 days after operative procedure - Infection involved only skin and subcutaneous tissue of incision - *At least one of the six organisms identified from an aseptically-obtained specimen from the superficial incision or subcutaneous tissue   Deep incisional SSI (Patient who had all of the following:)   - Infection occurred within 30 or 90 days after operative procedure^†^ - Infection involved deep soft tissue of the incision (i.e. fascial and muscle layer) - *At least one of the six organisms cultured from an aseptically-obtained specimen from the deep incision **and** fever (>38˚C) or/and localized pain or tenderness   Organ/Space SSI (Patient who had all of the following:)   - Infection occurred within 30 or 90 days after operative procedure^†^ - Infection involved any part of the body deeper than the fascial/muscle layers, that was opened or manipulated during the operative procedure - *At least one of the six organisms cultured from an aseptically-obtained fluid or tissue in the organ/space |
| Urinary tract infection (UTI) | Patient who had at least one of the following:   - ≥1 of these symptpms: fever (>38˚C), Suprapubic tenderness**, Costovertebral angle pain or tenderness**, urinary frequency***, urinary urgency***, dysuria*** - If patient was ≤1 year old, at least ONE of these symptoms: fever (>38˚C), hypothermia (<36˚C), apnea**, bradycardia**, lethargy**, vomiting**, suprapubic tenderness**   AND  *At least one of the six organisms cultured from urine specimen and the organism(s) was of ≥10^5^CFU/ml. |
| Other infections (OTH) | Skin/Soft tissue infection  Patient had at least one of the following:   - Purulent drainage - Pustules - Vesicles - Boils (excluding acne)   **AND**  at least two of the following symptoms: localized pain or tenderness, swelling, warmth, or redness  **AND**  *At least one of the six organisms cultured from aspirate or drainage from affected site  Other infections:  Patient who had clinical specimens collected from a sterile site culture positive for at least one of the six organisms* The sterile sites and affected sites included:   - CSF - Purulent drainage/material (from eye, ear canal, oral cavity, reproductive tract, etc.) - Fluid (joint fluid, peritoneal fluid, pericardial fluid, etc.) - Abscess - Tissues (lung tissue, brain tissue, spinal epidural, disc space, heart valve, bone, etc.) |
| More than one types of infection (>1 type of infections) | Patient who had two or more types of infection including LRTI, SSI, UTI and OTH as defined above with at least one of the six organisms*.  **OR**  **Secondary BSI**  Patient had at least one of the following:   - A BSI patient with another type of infection (LRTI, SSI, UTI or OTH as defined above) with at least one matching organism. - A BSI patient who had all of the criteria for another type of infection (LRTI, SSI, UTI or OTH as defined above) fulfilled except that none of the organisms were identified from the relevant clinical specimens |

* The bacteria under evaluation were *Staphylococcus aureus*, *Enterococcus* spp., *Escherichia* *coli*, *Klebsiella* *pneumoniae*, *Pseudomonas* *aeruginosa* and *Acinetobacter* spp..

** With no other documented causes

*** These symptoms (in grey) were not used when catheter is in place.

**Appendix 2.** Confounding factors defined based on the directed acyclic graph

| Confounding factor | Definition | Type of variable |
| --- | --- | --- |
| Admission ward | Responsible medical specialty at time of admission.  (Proxy for the patient characteristics and the severity of underlying illness at time of admission)* | Categorical variable  1= Non-ICU medical wards (adult/paediatrics/neonate)  2= Non-ICU surgical wards (adult/paediatrics/neonate)  3= Non-ICU haematology/oncology  (adult/paediatrics)  4= Non-ICU obstetrics/gynaecology  5= All ICUs |
| SOFA score on admission | The patients scores ranging from 0 to 24 from the Sequential Organ Failure Assessment (SOFA) score within 48 hours before or at time of admission.  (Proxy for the patient characteristics and the severity of underlying illness at time of admission)* | Continuous variable  0 or Positive integer |
| Transferred from other hospitals | Indicate whether patients were transferred from other hospitals.  (Proxy for the patient characteristics and the severity of underlying illness at time of admission)* | Binary variable  0= Not transferred from other hospitals  1= Transferred from other hospitals |
| Charlson Comorbidity Index (CCI) on admission** | A weight index based on specified comorbid conditions; including congestive heart failure, myocardial infarction, peripheral vascular disease, chronic obstructive pulmonary disease, ulcer disease, liver diseases, renal disease, connective tissue disease, cerebrovascular disease, hemiplegia or paraplegia, dementia, lymphoma, leukemia, tumor, diabetes, recipient of organ transplant, and HIV infection  (Proxy for the patient characteristics and the severity of underlying illness at time of admission)* | Categorical variable  0= score is 0  1= score is 1  .  .  .  8= score is 8 or above |
| Gender | Female or male  (Proxy for the patient characteristics and the severity of underlying illness at time of admission)* | Binary variable  0= female  1= male |
| Age | Age in year  (Proxy for the patient characteristics and the severity of underlying illness at time of admission)* | Continuous variable |
| Exposure to invasive device | Indicate whether or not patient had invasive device for at least 48 hours on the first date of culture-positive specimen collection of the HAI under evaluation. Invasive device referred to either peripheral-line catheter, central-line catheter, urinary-catheter, ventilator, or umbilical catheter.  (Proxy for the risks of acquiring AMR infections during hosptialisation)* | Binary variable  1= on invasive device for at least 48 hours  0= if otherwise |
| Cumulative days of exposure to parenteral antibiotics | Total number of days on antibiotics prior to the first date of culture-positive specimen collection of the HAI under evaluation.  (Proxy for the risks of acquiring AMR infections during hosptialisation)* | A spline function with 3 knots at 1, 8, and 14 days was applied to the continuous variable to relax the linearity assumption. |
| Length of hospitalisation prior to the infection | Total number of days being hospitalised prior to the first date of culture-positive specimen collection of the HAI under evaluation.  (Proxy for the risks of acquiring AMR infections during hosptialisation)* | A spline function with 5 knots at 4, 7, 11, 19, and 31 days was applied to the continuous variable to relax the linearity assumption. |
| Turnaround time for bacterial culture result | Total number of days between when specimen was collected and the results (including antimicrobial susceptibility test results) were reported  (Proxy for the bacterial load)* | Binary variable  0= Less than or equal to 3 days  1= more than 3 days |

*Detailed description on statistical analysis and confounders are in Appendix 3 and in Figure 1 (directed acyclic graph). **Charlson Comorbidity index was calculated based on previously published guideline (Table below):

| Condition | Score |
| --- | --- |
| Acute myocardial infarction | 1 |
| Congestive heart failure | 1 |
| Peripheral vascular disease | 1 |
| Cerebral vascular accident | 1 |
| Dementia | 1 |
| Chronic obstructive pulmonary disease | 1 |
| Connective tissue disorder | 1 |
| Peptic ulcer | 1 |
| Liver disease | 1 |
| Severe liver disease | 3 |
| Diabetes | 1 |
| Diabetes complications | 2 |
| Paraplegia | 2 |
| Renal disease | 2 |
| Tumor | 2 |
| Metastatic solid tumor | 3 |
| HIV | 6 |

**Appendix 3. Supplementary methods and results**

**Supplementary methods**

**Study design**

We prospectively enrolled patients who were admitted to Sunpasitthiprasong Hospital, a 1,201-bed tertiary hospital in Ubon Ratchathani, Northeast Thailand. The most commonly used parenteral antibiotics in this hospital were ceftriaxone, carbapenems and ceftazidime.^1^ All wards and patients of all age were screened. The inclusion criteria were patients, regardless of age or ward at which treatment was received, who developed BSI, LRTI, surgical-site infection (SSI), urinary tract infection (UTI) or other specific types of infections (OTH) after hospitalization for more than 48 hours with clinical specimens culture-positive for *Staphylococcus aureus*, *Enterococcus* spp., *Escherichia coli*, *Klebsiella pneumoniae*, *Pseudomonas aeruginosa*, or *Acinetobacter* spp.. We focused on these particular bacteria because they were in the 2015 global priority list of AMR bacteria from the WHO^2^ and are of clinical importance in hospitals in the Southeast Asian region.^3^ Specifically, we analyzed *A. baumannii* of *Acinetobacter* species and *E. faecium* and *E. faecalis* of *Enterococcus* species. Patients who had an onset of signs and symptoms of the current infections within 48 hours of hospitalization were excluded. Only the first episode of the first HAI under evaluation for each patient was included. Protocol training and a “dry-run” were performed between January and February 2018, before the surveillance study was rolled out between 1^st^ March 2018 and 29^th^ February 2020.

**Ethical approvals**

Written informed consent was obtained from participants over 18 years old, before enrollment. For all patients who were under 18 years old at the time of enrollment, written informed consent was obtained from their guardians. In addition, for patients between age 7 and 13 years informed assent were obtained from the patients, and for patients between 13 and 18 years informed consents were obtained from the patients.

**Laboratory procedure**

Throughout the study, blood culture sampling, bacterial culture, species identification and antimicrobial susceptibility testing (AST) were performed by the microbiology laboratory of the study hospital as per routine practices using standard methodologies provided by Department of Medical Science, Ministry of Public Health (MoPH), Thailand.^4-6^ The interpretation of the antibiotic disk diffusion method was based on Clinical and Laboratory Standards Institute (CLSI) guidelines.^6^

In general, at the study hospital, the decision in requesting a clinical sample to be collected for bacterial culture was made by the physician based on clinical judgements. For instance, when the doctor suspected an episode of bloodstream infection, venipuncture was performed by the nurse to collect blood sample. In short, blood specimens (5-10 mL if the patient is an adult and 1-3 ml if patient is a child) were collected in BD BACTEC blood culture bottles and process using the BD BACTEC automated system (Becton-Dickinson, Sparks, MD, USA). Other types of clinical specimen were collected in sterile containers and then transported to the laboratory for bacterial culture. During the study period, identifications and antimicrobial susceptibility testing (AST) of the bacteria isolated from blood and sterile-site specimens were routinely performed using VITEK 2 system (bioMérieux). Identification and AST of the bacteria isolated from non-sterile site specimens were routinely performed using biochemical testing and the disk diffusion method. The interpretation of the disk diffusion method was based on Clinical and Laboratory Standards Institute (CLSI) guidelines. All microbiology test results were then input and stored in MLAB program every evening.^7^ Then the microbiology data were exported in MS Excel format and sent to the hospital infection control nurse team and the study team on a daily basis throughout the study period.

**Outcome**

The primary outcome was mortality within 28 days from the first date of culture-positive specimen collection of the HAI under evaluation. On day 28, we contacted the patients who were no longer hospitalized (i.e. discharged before Day 28). Those who were lost to follow-up were assessed using the hospital admission data including the type of discharge and health status when discharged. Patients who were lost to follow-up and discharged against physician’s advice without an improvement in their health condition were considered as having died within 28 days of the first culture-positive specimen date.

**Statistical analysis**

**Estimate excess mortality attributable to AMR infections**

In this study, AMS infections were used as the comparator to estimate the impact of AMR infections on 28-day mortality. The excess risk of mortality and mortality attributable to AMR infections was estimated as described elsewhere.^8^ In brief, the observed mortality was compared with the counterfactual outcome of AMS infections. The excess risk of mortality is defined as the mortality in the study cohort that would be prevented if patients had AMS infections, adjusted for pre-defined confounders. This is calculated from the absolute difference between the expected mortality if all were AMS infections and the expected mortality if all were AMR infections. Population attributable fraction (PAF) is defined as the proportional reduction in population mortality that would be prevented if all patients had AMS infections, adjusted for pre-defined confounders.

$$Excess risk of mortality attributable to AMR= Pr\left[ Y^{AMR}=1 \right]-Pr\left[ Y^{AMS}=1 \right]$$

$$PAF of mortality attributable to AMR=\frac{Pr\left[ Y=1 \right]-Pr\left[ Y^{AMS}=1 \right]}{Pr\left[ Y=1 \right]}$$

[1]

where *Pr*[*Y*=1] was the observed mortality and *Pr*[*Y^AMS^*=1] was the mortality that would have been observed if everyone with HAI infections in this cohort had AMS infections. The inputs for the PAF were the observed number of all deaths, which is the crude mortality of hospital-acquired infection in our cohort, and the estimated number of deaths had the all patients have had AMS infections rather than AMR infections.^8-12^ The latter is estimated from the final model adjusted for the key confounders identified using the DAG in Appendix 4.

Firstly, a set logistic regression models were used to estimate the odds ratio for 28-day mortality of AMR infections compared to AMS infections, adjusted for the causative bacteria and types of infection. Secondly, an interaction term between the causative bacteria and AMR infections was added to take account of the varying effect of AMR infections of different causative bacteria on 28-day mortality.

Multivariable logistic regression models were built based on a direct acyclic graph (DAG). The pre-defined confounders between AMR or AMS infections and mortality were (a) the severity of underlying illness and patient characteristics on the hospitalisation day, (b) risks of acquiring AMR infections during the hospitalisation, and (c) bacterial load (Figure 1; detailed descriptions for each potential confounder were in Appendix 2). Firstly, the severity of underlying illness and patient characteristics on the hospitalisation day could represent the potential exposure to various level of contacts with healthcare workers and to invasive procedures, which were factors that influenced the probability of the patient being infected by AMR or AMS bacteria. The proxy variables included the Charlson Comorbidities Index (CCI) on admission, Sequential Organ Failure Assessment (SOFA) score on the day of hospitalisation, admission ward, transfer from another hospital, age and gender (Appendix 2). Secondly, a set of parameters were used to represent the risks of acquiring AMR infections during the hospitalisation, including duration of hospital stay, duration of antibiotic treatment, and exposure to invasive device prior to the first date of culture-positive specimen collection. These were potential drivers of acquiring AMR infections after the hospital admission. These drivers could involve within-host transmission dynamic of AMR and AMS bacteria which may determine the dominate strain of bacteria and isolating the bacteria from clinical specimen.^13^ Thirdly, the bacterial load could determine development of infection and probability that the diagnostic tests (i.e. bacterial culture) would be positive, chance of treatment success and consequently patient survival. The proxy variable used in the study to represent bacterial load was the number of days between the date of specimen collection and the date on which microbiology culture and AST results were reported.

We considered that SOFA score on the first date of culture-positive specimen collection and antibiotic treatment after the first date of culture-positive specimen collection were on the causal pathway to the 28-day mortality outcome. Therefore, those two variables were not included in the models.

Likelihood ratio test was performed to assess the optimal forms of each continuous variable to include into the model. For example, likelihood ratio test was performed to compare a model with SOFA score on admission as a categorical variable against a model with SOFA score on admission as a continuous variable with the linear assumption. The result of the likelihood ratio test showed that the two models fitted the dataset equally well and that the estimated excess mortality and deaths attributable to AMR were not different; therefore, SOFA score on admission was included as continuous variable in the final model. Similar tests were performed independently for CCI on admission, age, cumulative days of exposure to parenteral antibiotics, and length of hospitalization prior to the infection. In the final model, SOFA score on admission and age were included as continuous variables. CCI on admission was included as a categorical variable, as the linear assumption did not hold and 95% of the patients had a score less than nine. The linearity assumption was relaxed for length of hospitalization prior to the infection on outcome, using restricted cubic splines with five knots at the 5^th^, 25^th^, 50^th^, 75^th^ and 95^th^ percentiles. The linearity assumption was also relaxed for cumulative days of exposure to parenteral antibiotics, using restricted cubic splines with three knots at the 25^th^, 50^th^ and 75^th^ percentiles.

We also explored the estimated excess mortality attributable to AMR infections when models were adjusted for a subset of confounders, which could be retrievable from routinely collected electronic data in some resource-limited settings. The variables considered included admission ward, direct admission to ICU, gender, age, CCI on admission, and duration of hospital stay prior to the first date of culture-positive specimen collection (results are presented in Appendix 9). The CCI score could be defined by using data from International Classification of Disease (ICD)-9 or ICD-10 coding, and duration of hospital stay prior to the first date of culture-positive specimen collection could be calculated from hospital admission date and specimen collection date.

**Sensitivity analyses**

Sensitivity analyses was performed to assess the impact of the patients who lost to follow-up had on the estimated attributable mortality. The results of the sensitivity analyses are presented in Appendix 9. Firstly, the final model was performed on the cohort of only patients whose 28-day survival status was known (i.e. excluding those who lost to follow-up). Secondly, the final model was performed on the full cohort and we assumed that all patients who lost to follow-up survived. Thirdly, sensitivity analyses were done to assess the impact of *E. faecalis* and *E. faecium* on the estimated attributable mortality (Appendix 9).

**Results**

**Patient population**

The proportion of patients that were directly admitted to ICUs on the hospital admission was slightly higher in the AMR infection group (25.2%; [349/1,385]) compared to AMS infection group (23.7% [156/658]). There were substantial differences in antibiotic use between the two groups of patients. The proportion of patients who had at least one dose of parenteral antibiotic prior to the first date of the culture-positive specimen collection was 90.6% (1,255/1,385) in the AMR infection group, and 84.5% (n=556/658) in the AMS infection group. The cumulative days of parenteral antibiotic exposure prior to the first date of the culture-positive specimen collection was longer in the AMR infection group compared to the AMS infection group (median: 9 vs 3 days) (Table 1). Violin plots below show the distribution of the continuous variables by types of infections for patients with AMS and AMR infections.

**Violin plots to show distribution of age, SOFA score on admission, Charlson’s comorbidity score on admission, length of hospitalisation prior to the infection, and cumulative days of exposure to parenteral antibiotics by types of infection for patients with antimicrobial-susceptible (AMS) and antimicrobial-resistant (AMR) infections.**


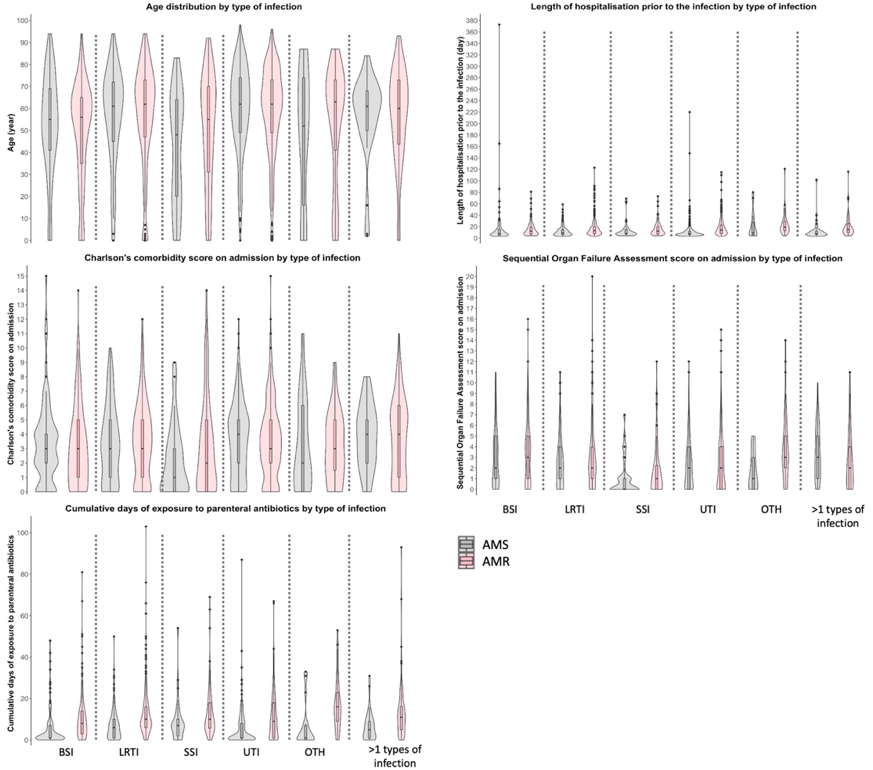


**Causative bacteria**

Of all monomicrobial infections, the proportion of 3GCREC and 3GCRKP among infections with *E. coli* and *K. pneumoniae* were 65.1% (188/289) and 78.5% (267/340), respectively. Amongst infections with *E. coli*, the proportion of carbapenem-resistant *E. coli* was 14.9% (43/289). Amongst infections with *K. pneumoniae*, the proportion of carbapenem-resistant *K. pneumoniae* was 16.5% (56/340).

**Patient characteristics by type of infections**

There were 35 patients with central-line associated BSI (out of 289 patients with BSI; 12.1%). The majority of LRTI and UTI patients had ventilator-associated pneumonia (66.2%; n=409/618) and catheter-associated UTI (71.6%; n=570/796), respectively. The majority of SSI patients had superficial incisional SSI (73.6%; 114/155), followed by organ/space SSI (19.4%; 30/155) and deep incisional SSI (7.1%; 11/155).

Exposure to antibiotic treatment and duration of hospitalization prior to the first date of culture-positive specimen collection varied by types of infection. The median of the cumulative on parenteral antibiotic treatment prior to the first date of culture-positive specimen collection was highest among patients with OTH (13 days; IQR: 4-23 days) and shortest among patients with BSI (6 days; IQR: 1-13 days) and UTI (6 days; IQR: 1-14). The median hospitalization days prior to the first date of culture-positive specimen collection was highest among those with OTH (median 19 days [IQR: 11-29 days]) and shortest among those with BSI (median 10 days [IQR: 6-18]). The median of CCI score on the hospital admission was 3 (IQR: 1-5) both in patients with AMR infections and those with AMS infections.

**Observed mortality**

The highest crude 28-day mortality was among patients with more than one type of infection (54 deaths out of 177 patients; 46.2%), followed by BSI (114 deaths out of 289 patients; 39.5%) and LRTI (231 deaths out of 618 patients; 37.4%) (Appendix 6). The lowest crude 28-day mortality was among patients with SSI (20 deaths out of 155 patients; 12.9%). Crude cumulative hazard of mortality of patients with AMS and AMR infections is showed below.

The final model suggested that the odds of mortality increased with increased CCI score, SOFA score on hospital admission, and the cumulative days of exposure to parenteral antibiotics (Appendix 10).

**Sensitivity analysis**

Sensitivity analyses were done to assess the impact of AMR on mortality i) with adjustment for a subset of confounders, ii) without adjustment for confounders, iii) under different assumptions on the outcome of the 39 cases who were lost to follow-up, and iv) excluding patients infected with *E. faecalis* and *E. faecium*. The results showed that excess mortality attributable to AMR infections would be overestimated in models adjusted with a subset of confounders or without adjustment for confounders (Appendix 9). The estimated population attributable fraction of AMR infections ranged from 14.4% to 14.7% based on different assumptions used for those 39 cases (Appendix 9). When both *E. faecalis* and *E. faecium* infections were excluded from the analysis, the estimated population attributable fraction of AMR infections was 14.1%, which was comparable to the findings from the final model (Appendix 9).

**Discussion**

Cassini et al^14^ used parameters from studies comparing AMR infections with AMS infections and no infection, but prioritized the no infection comparator. Parameters from the GBD study^15^ was from the comparison between AMR and AMS infections using a counterfactual approach. Both no infection and AMS counterfactuals can provide policy-relevant information about future interventions designed to control spread of AMR infections.^16^ Our estimates are not comparable with the attributable mortality of AMR infections reported from Morocco^17^ because our estimated excess mortality (7.7 percentage points) and PAF (16.3%) were derived from a statistical model using a counterfactual approach whereas the study from Morocco reported the proportion of the observed number of patients who had MDR bacterial infections and died over the total number of patients included in the study (12%; 58/479).

There are several limitations to this study, as indeed there are many studies addressing this important question. First there are important residual confounding factors. Antibiotic use before hospital admission is common, but often not recorded. Factors (such as immunocompromise) increase both the risk of infection and death, particularly in the elderly. Future study to systematically record and adjust for the prescription of corticosteroid would be useful to reduce residual confounding. Moreover, the assumption of time-fixed exposure (AMR infection) was made and could be relaxed in future studies. Second, some important pathogens (*S. aureus*, *Acinetobacter* spp.) could colonize skin and could be incidental in blood cultures. Colonization of the upper airway with Gram-negative pathogens is usual in patients with protracted intubation. Sputum or bronchial aspirate cultures commonly contain AMR organisms, but they may not be the ones causing LRTI. Strict case definition and adequate radiographic review is often required to support the differentiation between a colonizer and a cause of infection. However, such information cannot easily be extracted retrospectively. For this reason, our prospective design using standardized definitions of infections was necessary. In our study, we have observed high crude mortality in *A. baumanii* LRTI, which indirectly suggests that the case identification process used for infections was likely specific. Protracted urinary catheterisation commonly leads to bacterial colonization. Further study using advanced technology that detects host responses such as matrix-assisted laser desorption/ionization time-of-flight mass spectrometry and gene expression profiling could be used to support diagnosis of infection. However, the usage is such technology is currently limited. Third, excess mortality and deaths attributable to AMR infections compared to AMS infections represents the lower bound of burden of AMR infections, and may underestimate potential impacts of future interventions and preventive measures against AMR infections.^18,19^ An additional analysis to estimate the excess deaths of AMR infections compared to patients with no infections will be needed to inform the upper bound of burden of AMR infections.^18,19^ Fourth, as the study was performed in a single study site, the results may not be generalizable to other settings where patient characteristics, patient management, antibiotic availability and usage, and practices of collecting clinical samples for microbiology culture (including frequency and timing) may differ from the study hospital. However, the study design can be readily adapted and applied in other LMICs with a relative high sustainability to produce detailed information to support target organism(s) and type(s) of infection to intervene. Subsequent episodes of HAI prior 28-day outcome could be an important mediator of the first episode of AMR and 28-day outcome, and further studies with appropriate analysis could be done to quantify the impact of subsequent episodes on patient outcome. Finally, the appropriateness of antibiotic use could be an important mediator of AMR infections and mortality. Further studies with appropriate adjustment for immortal-time bias and time-varying confounders could be done to disentangle the relationships and improve the understanding of the mechanism underlying the impact of AMR.^20^

**Crude cumulative hazard of mortality of patients with antimicrobial-susceptible (AMS) and antimicrobial-resistant (AMR) infections.**


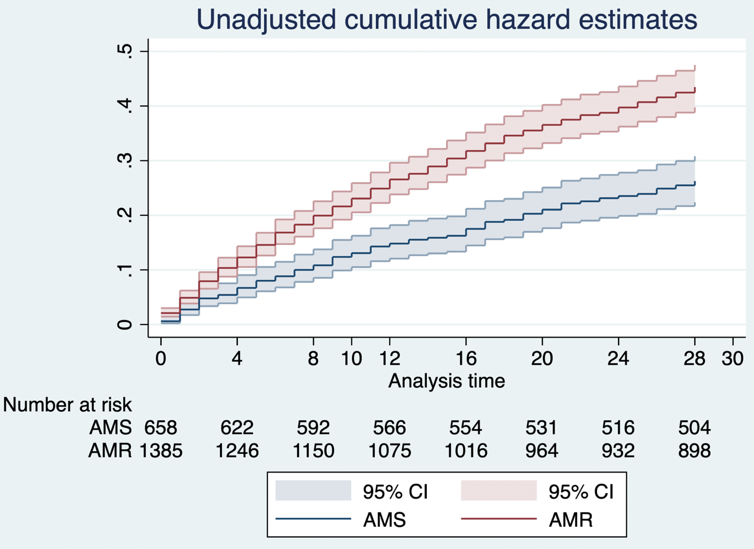


**References**

1. Lim C, Hantrakun V, Teerawattanasook N, Srisamang P, Teparrukkul P, Sumpradit N, Turner P, Day NPJ, Coope BS, Peacock SJ, Limmathuratsakul D. J Infect, **2021**;82(3):355-362.

2. World Health Organization. Global priority list of antibiotic-resistant bacteria to guide research, discovery, and development of new antibiotics, **2017**.

3. Hongsuwan M, Srisamang P, Kanoksil M, et al. Increasing incidence of hospital-acquired and healthcare-associated bacteremia in northeast Thailand: a multicenter surveillance study. PLoS One, **2014**;9(10):e109324.

4. Ministry of Public Health, Thailand. Laboratory manual for bacteriology. **2014**. <http://narst.dmsc.moph.go.th/manuals/Idenbook.pdf> Accessed 21 June 2021.

5. Ministry of Public Health, Thailand. Manual for the Laboratory identification and antimicrobial susceptibility testing of bacterial pathogens of publich health importance in the developing world. **2003**. <http://narst.dmsc.moph.go.th/manuals/AMRmanual_chapters1-5.pdf> Accessed 21 June 2021.

6. National Committee for Clinical Laboratory Standards. Analysis and Presentation of Cumulative Antimicrobial Susceptibility Test Data, 4th Edition. **2014**.

7. Sriboonsong S, Boonchoo L. Automatic antimicrobial susceptibility testing system. 2007; Berlin, Heidelberg: Springer Berlin Heidelberg; 2007. p. 2501-4.

8. Hernán MA, Robins JM. Causal Inference: What If: Boca Raton: Chapman & Hall/CRC; 2020

9. von Cube M, Timsit JF, Schumacher M, Motschall E, Schumacher M. Quantification and interpretation of attributable mortality in core clinical infectious disease journals. Lancet Infect Dis, **2020**;20(12):e299-e306.

10. Rockhill B., Newman B., and Weinberg C. Use and misuse of population attributable fractions. Am J Public Health, **1998**;88(1):15-19.

11. Greenland S, Robins JM. Conceptual problems in the definition and interpretation of attributable fractions. Am J Epid, **1988**; 128(6): 1185-97.

12. Hanley JA. A heuristic approach to the formulas for population attributable fraction. Theory and methods, **2001**;55:508-514.

13. Davies NG, Flasche S, Jit M, Atkins KE. Within-host dynamics shape antibiotic resistance in commensal bacteria. *Nat Ecol Evol* **2019**; 3(3): 440-9.

14. Cassini A, Hogberg LD, Plachouras D, et al. Attributable deaths and disability-adjusted life-years caused by infections with antibiotic-resistant bacteria in the EU and the European Economic Area in 2015: a population-level modelling analysis. Lancet Infect Dis, **2019**;19(1):56-66.

15. Antimicrobial Resistance Collaborators. Global burden of bacterial antimicrobial resistance in 2019: a systematic analysis. Lancet, **2022**; online first <https://www.thelancet.com/journals/lancet/article/PIIS0140-6736(21)02724-0/fulltext>.

16. de Kraker MEA, Lipsitch M. Burden of Antimicrobial Resistance: Compared to What? Epidemiol Rev, **2021**.

17. El Mekes A, Zahlane K, Ait Said L, Tadlaoui Ouafi A, Barakate M. The clinical and epidemiological risk factors of infections due to multi-drug resistant bacteria in an adult intensive care unit of University Hospital Center in Marrakesh-Morocco. J Infect Public Health, **2020**;13(4):637-43.

18. World Health Organization. GLASS method for estimating attributable mortality of antimicrobial resistant bloodstream infections, **2020**.

19. de Kraker MEA, Lipsitch M. Burden of Antimicrobial Resistance: Compared to What? Epidemiol Rev, **2021**.

20. Lim C, Mo Y, Teparrukkul P, Hongsuwan M, Day NPJ, Limmathurotsakul D, Cooper BS. Effect of delays in concordant antibiotic treatment on mortality in patients with hospital-acquired Acinetobacter species bacteraemia: emulating a target randomised trial with 13 year retrospecitve cohort. Am J Epidemiol, **2021**;190(11):2395-2404.

**Appendix 4.** Directed acyclic graph to illustrate the possible relationship between hospital-acquired bacterial infections and 28-day mortality of patients.


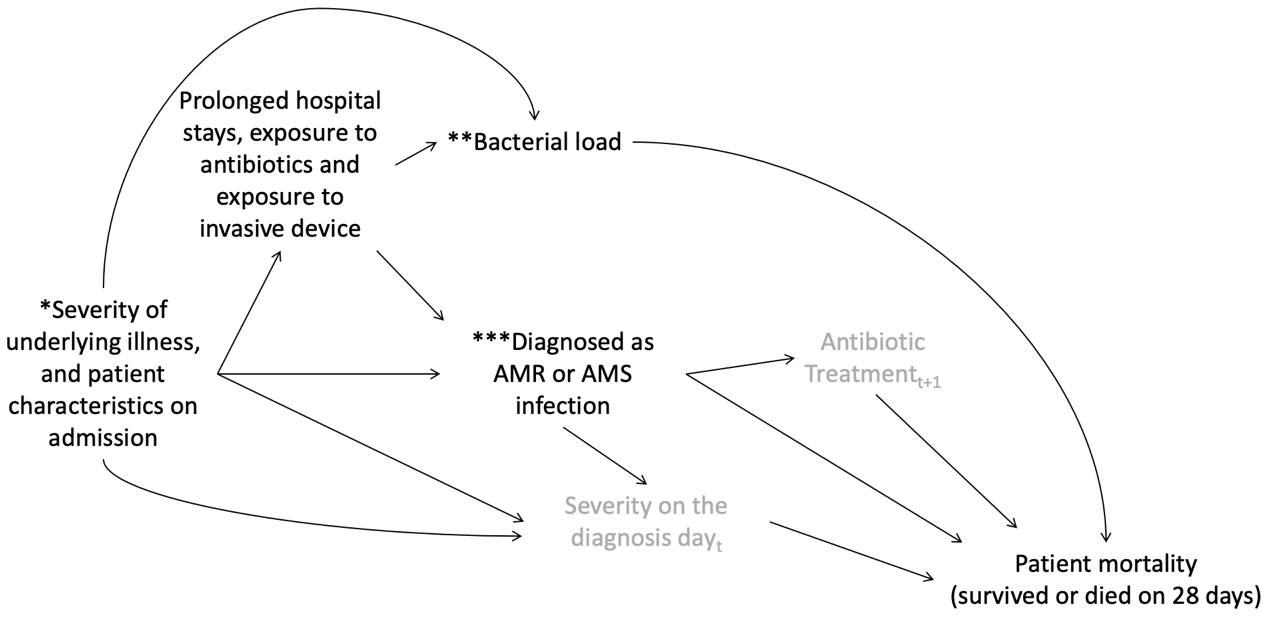


**Footnote:** Arrows indicate the direction of a potential causal relationship; for instance, the relationship between severity of underlying illnesses and the probability of AMR infection is represented by an arrow from “Severity of underlying illness” to “AMR or AMS infection”. *The proxy variables to represent the potential baseline confounders for the severity of underlying illnesses and patient characteristics included the Charlson Comorbidities Index score, department and ward type (ICU vs. non-ICU) to which patient was admitted on the day of hospitalization, gender, age, whether or not patient was referred from another hospital, and SOFA score recorded when patients were hospitalized. After hospital admission the level of exposure to AMR bacteria in the hospital environment is a potential confounder, which was represented by proxy variables including the duration of hospitalization, cumulative days of exposure to antibiotic treatment prior to specimen collection, and exposure to invasive device prior to the date the culture-positive specimen was collected. **The proxy variable used to represent bacterial load is the number of days it took to obtain the microbiology test results. ***Diagnosed AMR/AMS infections with the bacteria under evaluation. The events in grey color are those that occur after the onset of or are caused by infections (i.e., mediators between the type of infection and 28-day mortality).

**Appendix 5.** Characteristics of patients included in the analysis stratified by specific type of infections.

| **Characteristics** | **All patients (N=2,043)** | **BSI  (N=289)** | **LRTI  (N=618)** | **SSI**  **(N=155)** | **UTI  (N=796)** | **OTH  (N=68)** | **>1 type of infections (N=117)** |
| --- | --- | --- | --- | --- | --- | --- | --- |
| **Sex** |  |  |  |  |  |  |  |
| **Male** | 1,163 (56.9%) | 161 (55.7%) | 388 (62.8%) | 93 (60.0%) | 408 (51.3%) | 43 (63.2%) | 70 (59.8%) |
| **Age** |  |  |  |  |  |  |  |
| **≤0** | 84 (4.1%) | 20 (6.9%) | 31 (5.0%) | 12 (7.7%) | 9 (1.1%) | 8 (11.8%) | 4 (3.4%) |
| **1-4** | 22 (1.1%) | 3 (1.0%) | 7 (1.1%) | 2 (1.3%) | 7 (0.9%) | 1 (1.5%) | 2 (1.7%) |
| **5-14** | 42 (2.1%) | 6 (2.1%) | 11 (1.8%) | 7 (4.5%) | 13 (1.6%) | 4 (5.9%) | 1 (0.9%) |
| **15-24** | 102 (5.0%) | 17 (5.9%) | 28 (4.5%) | 18 (11.6%) | 33 (4.1%) | 3 (4.4%) | 3 (2.6%) |
| **25-34** | 90 (4.4%) | 18 (6.2%) | 25 (4.0%) | 9 (5.8%) | 32 (4.0%) | 1 (1.5%) | 5 (4.3%) |
| **35-44** | 159 (7.8%) | 30 (10.4%) | 39 (6.3%) | 13 (8.4%) | 62 (7.8%) | 3 (4.4%) | 12 (10.3%) |
| **45-54** | 281 (13.8%) | 42 (14.5%) | 88 (14.2%) | 20 (12.9%) | 108 (13.6%) | 8 (11.8%) | 15 (12.8%) |
| **55-64** | 413 (20.2%) | 68 (23.5%) | 109 (17.6%) | 25 (16.1%) | 170 (21.4%) | 12 (17.6%) | 29 (24.8%) |
| **65-80** | 640 (31.3%) | 66 (22.8%) | 209 (33.8%) | 38 (24.5%) | 264 (33.2%) | 24 (35.3%) | 39 (33.3%) |
| **≥81** | 210 (10.3%) | 19 (6.6%) | 71 (11.5%) | 11 (7.1%) | 98 (12.3%) | 4 (5.9%) | 7 (6.0%) |
| **Bacteria under evaluation** |  |  |  |  |  |  |  |
| ***Staphylococcus aureus*** | 69 (3.4%) | 36 (12.5%) | 9 (1.5%) | 5 (3.2%) | 8 (1.0%) | 10 (14.7%) | 1 (0.9%) |
| ***Enterococcus faecium*** | 182 (8.9%) | 11 (3.8%) | NA | 6 (3.9%) | 160 (20.1%) | 3 (4.4%) | 2 (1.7%) |
| ***Enterococcus faecalis*** | 160 (7.8%) | 10 (3.5%) | NA | 10 (6.5%) | 134 (16.8%) | 2 (2.9%) | 4 (3.4%) |
| ***Escherichia coli*** | 289 (14.1%) | 61 (21.1%) | 13 (2.1%) | 33 (21.3%) | 164 (20.6%) | 1 (1.5%) | 17 (14.5%) |
| ***Klebsiella pneumoniae*** | 340 (16.6%) | 66 (22.8%) | 144 (23.3%) | 25 (16.1%) | 84 (10.6%) | 12 (17.6%) | 9 (7.7%) |
| ***Pseudomonas aeruginosa*** | 221 (10.8%) | 22 (7.6%) | 75 (12.1%) | 12 (7.7%) | 101 (12.7%) | 6 (8.8%) | 5 (4.3%) |
| ***Acinetobacter baumannii*** | 401 (19.6%) | 69 (23.9%) | 211 (34.1%) | 31 (20.0%) | 62 (7.8%) | 13 (19.1%) | 15 (12.8%) |
| **Polymicrobial** | 381 (18.6%) | 14 (4.8%) | 166 (26.9%) | 33 (21.3%) | 83 (10.4%) | 21 (30.9%) | 64 (54.7%) |
| **Admission wards** |  |  |  |  |  |  |  |
| **Non-ICU Medical wards** | 715 (35.0%) | 96 (33.2%) | 202 (32.7%) | 13 (8.4%) | 332 (41.7%) | 34 (50.0%) | 38 (32.5%) |
| **Non-ICU Surgical wards** | 740 (36.2%) | 90 (31.1%) | 203 (32.8%) | 102 (65.8%) | 282 (35.4%) | 15 (22.1%) | 48 (41.0%) |
| **Non-ICU haematology/oncology** | 62 (3.0%) | 31 (10.7%) | 7 (1.1%) | 0 (0.0%) | 17 (2.1%) | 4 (5.9%) | 3 (2.6%) |
| **Non-ICU obstetrics/gynaecology** | 19 (0.9%) | 4 (1.4%) | 2 (0.3%) | 10 (6.5%) | 3 (0.4%) | 0 (0.0%) | 0 (0.0%) |
| **ICUs** | 507 (24.8%) | 68 (23.5%) | 204 (33.0%) | 30 (19.4%) | 162 (20.4%) | 15 (22.1%) | 28 (23.9%) |
| **Health status at time of admission** |  |  |  |  |  |  |  |
| **Charlson comorbidity index*** | 3 (1-5) | 3 (1-5) | 3 (1-5) | 2 (0-4) | 3 (2-5) | 3 (1-5) | 4 (2-6) |
| **Transferred from other hospitals** | 1,384 (67.7%) | 171 (59.2%) | 449 (72.7%) | 94 (60.6%) | 548 (68.8%) | 41 (60.3%) | 81 (69.2%) |
| **SOFA score*** | 2 (0-4) | 3 (1-5) | 2 (1-4) | 1 (0-2) | 2 (0-4) | 3 (1-5) | 2 (1-5) |
| **Parenteral antibiotic usage prior to the first date of culture-positive specimen collection** |  |  |  |  |  |  |  |
| **Exposure to parenteral antibiotics** | 1,811 (88.6%) | 270 (93.4%) | 581 (94.0%) | 143 (92.3%) | 654 (82.2%) | 56 (82.4%) | 107 (91.5%) |
| **Cumulative days of exposure to parenteral antibiotics*** | 8 (1-14) | 6 (1-13) | 9 (5-15) | 8 (5-16) | 6 (1-14) | 13 (4-23) | 9 (4-15) |
| **Health status on the first date of first culture-positive specimen collection** |  |  |  |  |  |  |  |
| **In the ICU** | 946 (46.3%) | 120 (41.5%) | 413 (66.8%) | 56 (36.1%) | 267 (33.5%) | 20 (29.4%) | 70 (59.8%) |
| **SOFA score*** | 1 (0-4) | 3 (0-6) | 2 (1-4) | 0 (0-2) | 1 (0-3) | 1 (0-3) | 4 (1-7) |
| **Length of hospital stay** |  |  |  |  |  |  |  |
| **Days in the hospital prior specimen collection*** | 11 (7-19) | 10 (6-18) | 12 (7-18) | 11 (7-19) | 11 (7-20) | 19 (11-29) | 14 (7-21) |
| **Turnaround time for bacterial culture result** |  |  |  |  |  |  |  |
| **≤3 days** | 1,747 (85.5%) | 93 (32.2%) | 589 (95.3%) | 138 (89.0%) | 757 (95.1%) | 64 (94.1%) | 106 (90.6%) |
| **Outcome** |  |  |  |  |  |  |  |
| **28-day mortality** | 643 (31.5%) | 114 (39.5%) | 231 (37.4%) | 20 (12.9%) | 202 (25.4%) | 22 (32.4%) | 54 (46.2%) |
| **Times to death*ˆ** | 10 (4-17) | 6 (2-12) | 9 (5-18) | 12 (10-15) | 12 (6-18) | 16 (6-23) | 6 (3-14) |
| **Length of hospital stay after the first date of culture-positive specimen collection in survivors*** | 17 (8-33) | 16 (7-31) | 24 (13-43) | 14 (8-33) | 13 (7-25) | 16 (5-28) | 21 (9-37) |

**Footnote:** AMS: antimicrobial-susceptible; AMR: antimicrobial-resistant; BSI: bloodstream infection; LRTI: lower-respiratory tract infection; SSI: surgical-site infection; UTI: urinary-tract infection; OTH: other infection; “>1 type of infection” refers to patient who infections that occurred at two or more body; *Charlson comorbidity index, SOFA scores, cumulative days of exposure to parenteral antibiotic prior specimen collection among patients who had antibiotic treatment prior sample collection, and days in the hospital prior specimen collected are in median (interquartile range), and other data is in n (%). **Details on the classification is in Appendix A2. ˆStatistics were estimated for those who died.

**Appendix 6.** Crude 28-day mortality by causative bacteria and type of infection

|  | BSI, % (n) | LRTI, % (n) | SSI, % (n) | UTI, % (n) | OTH, % (n) | >1 type of infection, % (n) |
| --- | --- | --- | --- | --- | --- | --- |
| *S. aureus* | | | | | | |
| MSSA | 18.8% (6/32) | 22.2% (2/9) | 0% (0/5) | 14.3% (1/7) | 0% (0/10) | 0% (0/1) |
| MRSA | 25.0% (1/4) | NA | NA | 0% (0/1) | NA | NA |
| *E. faecium* | | | | | | |
| AMPSEfm | 0% (0/1) | NA | 0% (0/1) | 25.0% (1/4) | NA | NA |
| AMPREfm | 40.0% (4/10) | NA | 40.0% (2/5) | 32.1% (50/156) | 0% (0/3) | 50.0% (1/2) |
| *E. faecalis* | | | | | | |
| AMPSEfc | 22.2% (2/9) | NA | 0% (0/7) | 27.3% (30/110) | 100% (1/1) | 25.0% (1/4) |
| AMPREfc | 100% (1/1) | NA | 0% (0/3) | 50.0% (12/24) | 0% (0/1) | NA |
| *E. coli* | | | | | | |
| 3GCSEC | 17.6% (3/17) | 50.0% (2/4) | 16.7% (1/6) | 24.6% (17/69) | NA | 40.0% (2/5) |
| 3GCREC | 47.7% (21/44) | 66.7% (6/9) | 18.5% (5/27) | 21.1% (20/95) | 0% (0/1) | 58.3% (7/12) |
| *K. pneumoniae* | | | | | | |
| 3GCSKP | 16.7% (2/12) | 37.8% (14/37) | 0% (0/5) | 12.5% (2/16) | 0% (0/1) | 0% (0/2) |
| 3GCRKP | 44.4% (24/54) | 36.4% (39/107) | 10.0% (2/20) | 26.5% (18/68) | 45.5% (5/11) | 57.1% (4/7) |
| *P. aeruginosa* | | | | | | |
| CSPA | 29.4% (5/17) | 21.6% (11/51) | 11.1% (1/9) | 12.3% (7/57) | 0% (0/2) | 25.0% (1/4) |
| CRPA | 40.0% (2/5) | 25.0% (6/24) | 33.3% (1/3) | 15.9% (7/44) | 50.0% (2/4) | 0% (0/1) |
| *A. baumannii* | | | | | | |
| CSAB | 20.0% (3/15) | 25.7% (9/35) | 0.0% (0/5) | 14.3% (1/7) | 0.0% (0/1) | 100% (2/2) |
| CRAB | 63.0% (34/54) | 41.5% (73/176) | 7.7% (2/26) | 21.8% (12/55) | 33.3% (4/12) | 38.5% (5/13) |
| Polymicrobial* | | | | | | |
| AMS | 50.0% (1/2) | 39.4% (13/33) | 0% (0/5) | 24.0% (6/25) | 50.0% (1/2) | 36.4% (4/11) |
| AMR | 41.7% (5/12) | 42.1% (56/133) | 21.4% (6/28) | 31.0% (18/58) | 47.4% (9/19) | 50.9% (27/53) |

**Footnote:** *Polymicrobial refers to infections that caused by more than one of the six bacteria under this survey. MSSA: meticillin-susceptible *S. aureus*; MRSA: meticillin-resistant *S. aureus*; AMPSEfm: ampicillin-susceptible *E. faecium*; AMPREfm: ampicillin-resistant *E. faecium*; AMPSEfc: ampicillin-susceptible *E. faecalis*; AMPREfc: ampicillin-resistant *E. faecalis*; 3GCSEC: 3^rd^ generation cephalosporin-susceptible *E. coli*; 3GCREC: 3^rd^ generation cephalosporin-resistant *E. coli*; 3GCSKP: 3^rd^ generation cephalosporin-susceptible *K. pneumoniae*; 3GCRKP: 3^rd^ generation cephalosporin-resistant *K. pneumoniae*; CSPA: carbapenem-susceptible *P. aeruginosa*; CRPA: carbapenem-resistant *P. aeruginosa*; CSAB: carbapenem-susceptible *A.* *baumannii*; and CRAB: carbapenem-resistant *A.* *baumannii*.

**Appendix 7.** Burden of AMR infections stratified by causative bacterium, after adjusted for pre-defined confounders in the final model.

|  | **Number of cases** | **Number of deaths** | **Excess mortality attributable to AMR infections,**  **percentage points (95% CI)*** | **PAF of mortality attributable to AMR infections, % (95% CI)**** | **Excess deaths attributable to AMR infections, n (95% CI)** |
| --- | --- | --- | --- | --- | --- |
| **MSSA** | 64 | 9 | NA | NA | NA |
| **MRSA** | 5 | 1 | 0.9 (-22.7, 24.5) | 0.5 (-13.4, 12.7) | 0 (-1, 1) |
| **AMPSEfm** | 6 | 1 | NA | NA | NA |
| **AMPREfm** | 176 | 57 | 11.4 (-21.8, 44.5) | 34.5 (-204.9, 85.9) | 20 (-38, 78) |
| **AMPSEfc** | 131 | 34 | NA | NA | NA |
| **AMPREfc** | 29 | 13 | 18.2 (-1.2, 37.6) | 11.5 (-2.0, 23.3) | 5 (0, 11) |
| **3GCSEC** | 101 | 25 | NA | NA | NA |
| **3GCREC** | 188 | 59 | 5.2 (-5.5, 15.8) | 11.6 (-15.8, 32.4) | 10 (-10, 30) |
| **3GCSKP** | 73 | 18 | NA | NA | NA |
| **3GCRKP** | 267 | 92 | 6.1 (-5.5, 17.7) | 15.1 (-18.9, 39.3) | 16 (-15, 47) |
| **CSPA** | 140 | 25 | NA | NA | NA |
| **CRPA** | 81 | 18 | 5.9 (-5.0, 16.7) | 10.9 (-11.4, 28.7) | 5 (-4, 14) |
| **CSAB** | 65 | 15 | NA | NA | NA |
| **CRAB** | 336 | 130 | 12.5 (1.0, 24.0) | 29.1 (-3.5, 51.5) | 42 (3, 81) |
| **AMS polymicrobial infection^†^** | 78 | 25 | NA | NA | NA |
| **AMR polymicrobial infection^†^** | 303 | 121 | 1.9 (-9.3, 13.2) | 4.1 (-22.6, 25.0) | 6 (-27, 40) |
| **Total** | **2043** | **643** | **7.7 (2.2, 13.2)** | **16.3 (1.2, 29.1)** | **106 (30, 182)** |

**Footnote:** *Excess mortality is defined as the absolute mortality in the study cohort that would be prevented if the infections caused by the bacterium were AMS infections, adjusted for the influence of confounding factors. **Population attributable fraction (PAF) of mortality attributable to AMR infections is defined as the proportional reduction in population mortality that would be prevented if the infections caused by the bacterium were AMS infections, adjusted for the influence of confounding factors. ^†^Polymicrobial is defined as infections with more than one of the bacteria in the evaluation. MSSA: meticillin-susceptible *S. aureus*; MRSA: meticillin-resistant *S. aureus*; AMPSEfm: ampicillin-susceptible *E. faecium*; AMPREfm: ampicillin-resistant *E. faecium*; AMPSEfc: ampicillin-susceptible *E. faecalis*; AMPREfc: ampicillin-resistant *E. faecalis*; 3GCSEC: 3^rd^ generation cephalosporin-susceptible *E. coli*; 3GCREC: 3^rd^ generation cephalosporin-resistant *E. coli*; 3GCSKP: 3^rd^ generation cephalosporin-susceptible *K. pneumoniae*; 3GCRKP: 3^rd^ generation cephalosporin-resistant *K. pneumoniae*; CSPA: carbapenem-susceptible *P. aeruginosa*; CRPA: carbapenem-resistant *P. aeruginosa*; CSAB: carbapenem-susceptible *A.* *baumannii*; and CRAB: carbapenem-resistant *A.* *baumannii.*

**Appendix 8.** Burden of AMR infections stratified by type of infection, after adjusted for pre-defined confounders in the final model.

|  | Number of cases | Number of deaths | Excess mortality attributable to AMR infections,  percentage points (95% CI)* | PAF of mortality attributable to AMR infections, % (95% CI)** | Excess deaths attributable to AMR infections, n (95% CI) |
| --- | --- | --- | --- | --- | --- |
| Bloodstream infections |  |  |  |  |  |
| AMS | 105 | 22 | NA | NA | NA |
| AMR | 184 | 92 | 8.4 (1.5, 15.4) | 14.8 (2.2, 25.7) | 16 (3, 28) |
| Lower-respiratory tract infections |  |  |  |  |  |
| AMS | 169 | 51 | NA | NA | NA |
| AMR | 449 | 180 | 7.8 (1.2, 14.4) | 15.9 (0.6, 28.8) | 35 (5, 65) |
| Surgical-site infections |  |  |  |  |  |
| AMS | 43 | 2 | NA | NA | NA |
| AMR | 112 | 18 | 3.8 (0.8, 6.7) | 20.4 (1.2, 35.8) | 4 (1, 8) |
| Urinary-tract infections |  |  |  |  |  |
| AMS | 295 | 65 | NA | NA | NA |
| AMR | 501 | 137 | 8.5 (0.5, 16.4) | 19.3 (-11.9, 41.8) | 42 (3, 82) |
| Other infections |  |  |  |  |  |
| AMS | 17 | 2 | NA | NA | NA |
| AMR | 51 | 20 | 5.9 (0.0, 11.7) | 15.7 (-1.8, 30.1) | 3 (0, 6) |
| >1 type of infections |  |  |  |  |  |
| AMS | 29 | 10 | NA | NA | NA |
| AMR | 88 | 44 | 5.9 (-1.7, 13.4) | 8.7 (-5.8, 21.1) | 5 (-1, 12) |
| Total | **2043** | **643** | **7.7 (2.2, 13.2)** | **16.3 (1.2, 29.1)** | **106 (30, 182)** |

**Footnote:** *Excess mortality is defined as the mortality in the study cohort that would be prevented if the infections of the specific type were AMS infections, adjusted for the pre-defined confounding factors. **Population attributable fraction (PAF) of mortality attributable to AMR infections is defined as the proportional reduction in population mortality that would be prevented if the infections of the specific type were AMS infections, adjusted for the influence of confounding factors.

**Appendix 9.** Estimated parameter values from different models

| Models* | Confounders adjusted in the model | Estimated causal risk difference between AMR infections and AMS infections,  percentage points (95% CI) | PAF of mortality attributable to AMR infections (95% CI)ˆ | Excess deaths attributable to AMR infections, n (95% CI) |
| --- | --- | --- | --- | --- |
| Final model (logistic regression model with the interaction term)** | Pre-defined confounders^†^ | 7.7 (2.2, 13.2) | 16.3% (1.2, 29.1%) | 106 (30, 182) |
| With adjustment for a subset of confounders or without adjustment for confounders | | | | |
| Logistic regression model with the interaction term** | A subset of pre-defined variables^††^ | 9.3 (3.9, 14.7) | 19.7% (5.4, 31.9%) | 129 (55, 203) |
| Logistic regression model with the interaction term** | Unadjusted | 10.9 (5.6, 16.2) | 23.8% (9.5, 35.9%) | 151 (77, 224) |
| Logistic regression model without the interaction term | Pre-defined confounders^†^ | 7.3 (2.3, 12.2) | 16.1% (4.4, 26.3%) | 100 (31, 170) |
| Logistic regression model without the interaction term | A subset of pre-defined variables^††^ | 8.7 (3.9, 13.6) | 19.2% (8.0, 29.0%) | 121 (54, 188) |
| Logistic regression model without the interaction term | Unadjusted | 10.0 (5.2, 14.7) | 21.8% (10.-31.5%) | 138 (72, 204) |
| Using different assumptions on the outcome of the 39 cases who were lost to follow-up | | | | |
| Final model on the cohort excluding 39 cases who lost to follow-up** | Pre-defined confounders^†^ | 6.9 (1.3, 12.4) | 14.5% (-1.1, 27.8%) | 95 (19, 172) |
| Final model on the full analysis cohort and assuming that 39 cases who lost to follow-up survived up to 28 days of infection** | Pre-defined confounders^†^ | 6.9 (1.4, 12.4) | 14.7% (-1.1, 28.1%) | 95 (19, 171) |
| Final model on the full analysis cohort and assuming that 39 cases who lost to follow-up died within 28 days of infection** | Pre-defined confounders^†^ | 7.0 (1.5, 12.5) | 14.4% (-0.3, 27.0%) | 97 (20, 173) |
| Excluding patients infected with *E. faecalis* and *E. faecium*. | | | | |
| Final model on the full analysis cohort and excluding patients infected with *E. faecalis* and *E. faecium*. | Pre-defined confounders^†^ | 6.0 (0.7, 11.5) | 14.1% (0.4, 26.0%) | 72 (8, 135) |
| Marginal structural model | | | | |
| Marginal structural model with inverse probability weighting | Pre-defined confounders^†^ | 9.0 (2.9, 15.0) | 24.7% (6.1, 40%) | 124 (40, 208) |

CI: confidence interval; *All models were adjusted for type of infection and causative bacteria. **An interaction term between the bacteria and AMR infections was included in the model. ^†^Pre-defined confounders adjusted in the model were admission ward, SOFA score on admission, transferred from other hospitals, CCI on admission, gender, age, exposure in invasive device, cumulative days of exposure to parenteral antibiotics, length of hospitalisation prior to the infection, and turnaround time for bacterial culture result. ^††^Selected variables adjusted in the model were confounders that could be captured in hospital admission records in a retrospective study design, and these were admission ward, gender, age, CCI on admission, and length of hospitalisation prior to the infection. **ˆ**Population attributable fraction (PAF) of mortality attributable to AMR infections is defined as the proportional reduction in population mortality that would be prevented if the infections of the specific type were AMS infections, adjusted for the influence of confounding factors.

**Appendix 10.** Multivariable logistic regression model with an interaction term for causative pathogen and resistance in patients who had hospital-acquired infections.

| Pre-defined confounders | Adjusted odds ratio |
| --- | --- |
| Bacteria under evaluation^1^ |  |
| *Staphylococcus aureus* |  |
| MSSA | 1.0 |
| MRSA | 1.1 (0.1, 8.0) |
| *Enterococcus faecium* |  |
| AMPSEfm | 1.0 |
| AMPREfm | 1.9 (0.2, 16.4) |
| *Enterococcus faecalis* |  |
| AMPSEfc | 1.0 |
| AMPREfc | 2.5 (1.0, 6.3) |
| *Escherichia coli* |  |
| 3GCSEC | 1.0 |
| 3GCREC | 1.3 (0.7, 2.4) |
| *Klebsiella pneumoniae* |  |
| 3GCSKP | 1.0 |
| 3GCRKP | 1.4 (0.7, 2.6) |
| *Pseudomonas aeruginosa* |  |
| CSPA | 1.0 |
| CRPA | 1.5 (0.7, 3.1) |
| *Acinetobacter baumannii* |  |
| CSAB | 1.0 |
| CRAB | 1.9 (1.0, 3.7) |
| Polymicrobial^†^ |  |
| AMS polymicrobial infection | 1.0 |
| AMR polymicrobial infection | 1.1 (0.6, 1.9) |
| Types of infection |  |
| BSI | 1.0 |
| LRTI | 0.8 (0.5, 1.2) |
| SSI | 0.3 (0.1, 0.5) |
| UTI | 0.4 (0.3, 0.7) |
| Others | 0.6 (0.3, 1.1) |
| MultipleSite | 1.0 (0.6, 1.7) |
| Admission wards |  |
| Non-ICU medical wards | 1.0 |
| Non-ICU surgical wards | 0.7 (0.5, 0.8) |
| Non-ICU haematology/oncology | 1.0 (0.5, 1.9) |
| Non-ICU obstetrics/gynaecology | 0.2 (0.02, 1.7) |
| ICUs | 0.5 (0.4, 0.7) |
| Sex |  |
| Female | 1.0 |
| Male | 1.0 (0.8, 1.2) |
| Age | 1.0 (1.0, 1.0) |
| Charlson comorbidity index (CCI) score |  |
| CCI score 0 | 1.0 |
| CCI score 1 | 2.1 (1.3, 3.5) |
| CCI score 2 | 1.6 (1.0, 2.5) |
| CCI score 3 | 2.3 (1.4, 3.8) |
| CCI score 4 | 2.6 (1.6, 4.4) |
| CCI score 5 | 4.0 (2.3, 6.9) |
| CCI score 6 | 3.9 (2.2, 6.9) |
| CCI score 7 | 2.8 (1.5, 5.3) |
| CCI score 8 or above | 4.2 (2.4, 7.4) |
| Transferred from other hospitals |  |
| Not transferred from other hospitals | 1.0 |
| Transferred from other hospitals | 0.9 (0.7, 1.1) |
| SOFA score on admission | 1.1 (1.1, 1.1) |
| Cumulative days of exposure to parenteral antibiotics* | 1.3 (1.0, 1.7) |
| Length of hospitalisation prior to the infection** | 0.9 (0.4, 1.8) |
| On invasive device for at least 48 hours |  |
| No | 1.0 |
| Yes | 1.1 (0.9, 1.4) |
| Turnaround time for bacterial culture result |  |
| ≤3 days *** | 1.0 |
| >3 days *** | 1.2 (0.8, 1.7) |

**Footnote:** *A spline function with 3 knots at 1, 8, and 14 days was applied to the cumulative days of exposure to parenteral antibiotics to relax the linearity assumption. **A spline function with 5 knots at 4, 7, 11, 19 and 31 days was applied to length of hospitalisation prior to the infection to relax the linearity assumption. ***Turnaround time for bacterial culture results was a proxy for bacterial load, which is a strong risk factors of mortality. ^†^Polymicrobial is defined as infections with more than one of the bacteria in the evaluation. MSSA: meticillin-susceptible *S. aureus*; MRSA: meticillin-resistant *S. aureus*; AMPSEfm: ampicillin-susceptible *E. faecium*; AMPREfm: ampicillin-resistant *E. faecium*; AMPSEfc: ampicillin-susceptible *E. faecalis*; AMPREfc: ampicillin-resistant *E. faecalis*; 3GCSEC: 3^rd^ generation cephalosporin-susceptible *E. coli*; 3GCREC: 3^rd^ generation cephalosporin-resistant *E. coli*; 3GCSKP: 3^rd^ generation cephalosporin-susceptible *K. pneumoniae*; 3GCRKP: 3^rd^ generation cephalosporin-resistant *K. pneumoniae*; CSPA: carbapenem-susceptible *P. aeruginosa*; CRPA: carbapenem-resistant *P. aeruginosa*; CSAB: carbapenem-susceptible *A.* *baumannii*; and CRAB: carbapenem-resistant *A.* *baumannii.* ^1^An interaction term between the causative bacteria and AMR infection was added to take account of the varying effect of AMR infection of different causative bacteria on 28-day mortality. The reported adjusted odds ratio is the impact of AMR on mortality by each causative bacteria; for instance, the adjusted odds ratio of mortality comparing MRSA against MSSA is 1.1 (0.1, 8.0).
